# Molecular mechanisms and functions of pyroptosis in sepsis and sepsis-associated organ dysfunction

**DOI:** 10.3389/fcimb.2022.962139

**Published:** 2022-07-29

**Authors:** Ri Wen, Yong-Ping Liu, Xiao-Xu Tong, Tie-Ning Zhang, Ni Yang

**Affiliations:** Department of Pediatrics, Pediatric Intensive Care Unit (PICU), Shengjing Hospital of China Medical University, Shenyang, China

**Keywords:** sepsis, pyroptosis, inflammation, cell death, sepsis-associated organ dysfunction

## Abstract

Sepsis, a life-threatening organ dysfunction caused by a dysregulated host response to infection, is a leading cause of death in intensive care units. The development of sepsis-associated organ dysfunction (SAOD) poses a threat to the survival of patients with sepsis. Unfortunately, the pathogenesis of sepsis and SAOD is complicated, multifactorial, and has not been completely clarified. Recently, numerous studies have demonstrated that pyroptosis, which is characterized by inflammasome and caspase activation and cell membrane pore formation, is involved in sepsis. Unlike apoptosis, pyroptosis is a pro-inflammatory form of programmed cell death that participates in the regulation of immunity and inflammation. Related studies have shown that in sepsis, moderate pyroptosis promotes the clearance of pathogens, whereas the excessive activation of pyroptosis leads to host immune response disorders and SAOD. Additionally, transcription factors, non-coding RNAs, epigenetic modifications and post-translational modifications can directly or indirectly regulate pyroptosis-related molecules. Pyroptosis also interacts with autophagy, apoptosis, NETosis, and necroptosis. This review summarizes the roles and regulatory mechanisms of pyroptosis in sepsis and SAOD. As our understanding of the functions of pyroptosis improves, the development of new diagnostic biomarkers and targeted therapies associated with pyroptosis to improve clinical outcomes appears promising in the future.

## 1. Introduction

Sepsis is a life-threatening organ dysfunction caused by a dysregulated host response to infection. It can progress to septic shock, which is accompanied by tissue and cell metabolic abnormalities (Singer et al., 2016). Epidemiological studies have found that there are approximately 18 million patients with sepsis worldwide every year with a mortality rate of 28–40%. The mortality rate of patients with septic shock is even higher (Walkey et al., 2015). Sepsis and septic shock are among the leading causes of death in patients in intensive care units worldwide, seriously threatening both life and health. Although diagnoses and treatments have improved for sepsis and septic shock, the current treatments are mainly symptomatic treatments and anti-infection therapy. Exploring the molecular mechanisms underlying sepsis is of great significance to develop novel therapeutic targets. The pathogenesis of sepsis is complicated, with inflammation and immune responses playing critical roles (Venet and Monneret, 2018; van der Poll et al., 2021). Recent studies have found that pyroptosis is involved in the regulation of inflammation and immune responses, suggesting a key role of pyroptosis in sepsis (He et al., 2021; Hsu et al., 2021; Miao et al., 2022).

As a form of pro-inflammatory programmed cell death, pyroptosis is characterized by the formation of pores on the cell membrane, cell swelling, and rupture accompanied by the release of abundant inflammatory factors and cellular contents (Cookson and Brennan, 2001; Galluzzi et al., 2018). Previous studies have shown that pyroptosis is involved in the occurrence and development of various inflammatory and infectious diseases (Liu et al., 2019; Xia et al., 2019; Zhou and Fang, 2019). Moderate pyroptosis can promote the release of pathogens by destroying infected cells, thereby recruiting immune cells to activate the host immune response, promoting the clearance of pathogens, and protecting the body against infections. However, the excessive activation of pyroptosis may adversely affect prognoses by aggravating the inflammatory response and cell or tissue damage. In recent years, an increasing number of studies have shown that pyroptosis plays an essential role in sepsis and sepsis-associated organ dysfunction (SAOD) (Chen et al., 2019; Martinez-Garcia et al., 2019).

In this review, we summarized the roles of pyroptosis in sepsis, highlighting more comprehensive molecular mechanisms regarding pyroptosis in sepsis from aspects of the potential roles of pyroptosis in septic organ dysfunctions, as well as first illustrating the factors that could influence the pyroptosis in sepsis. We also described the mechanisms that influence the onset of pyroptosis and the effects of pyroptosis on other forms of programmed cell death, providing evidence that pyroptosis plays a critical role in the process of sepsis.

## 2. Pyroptosis

Pyroptosis was originally known as inflammatory caspase-dependent cell death. However, with understanding of the gasdermins gene family deepens, pyroptosis is redefined as a gasdermins-mediated programmed cell death. Gasdermins, with the ability of forming pores on the membrane, are identified to be executors of pyroptosis. The family comprises six paralogous genes in humans: gasdermin A (GSDMA), gasdermin B (GSDMB), gasdermin C (GSDMC), GSDMD (GSDMD), GSDME (GSDME, DFNA5) and Pejvakin (PJVK, DFNB59). Mice carry genes GSDMA1-3, GSDMC1-4, GSDMD, GSDME and PJVK, but without GSDMD. Except for PJVK, Gasdermins comprise two domains, a N-terminal domain with pore-forming potential and a C-terminal inhibiting domain. Proteolytic cleavage between these two domains releases intramolecular inhibition and generates an active N-terminal (gasdermin-NT), allowing it to insert into the cell membrane and oligomerize to form pores that leads to pyroptosis (Liu et al., 2021). According to the Human Protein Atlas, each member of the gasdermins family exhibits distinct tissue expression (**Supplementary Figure 1**). Notably, during sepsis, the protein expression of GSDMA-NT, GSDMD-NT and GSDME-NT are reported to increase in sepsis-associated organ injuries, indicating that pyroptosis could play important roles in sepsis (Chen et al., 2021b; Teng et al., 2022). Depending on the inflammatory caspases involved, pyroptosis can be divided into the canonical pathway mediated by caspase-1, and the non-canonical pathway mediated by caspase-4, -5, or -11 (Kayagaki et al., 2015; Shi et al., 2015). In canonical and non-canonical pyroptosis pathway, inflammasome, a kind of macromolecular protein complex, plays a vital role as they provide a platform for the cleavage and maturation of inflammatory caspases. Notably, pyroptosis has been found to be also mediated by apoptotic caspase, granzyme, and elastase in recent years, which makes people have a more comprehensive understanding of pyroptosis (Wang et al., 2017; Sollberger et al., 2018; Zhou et al., 2020).

### 2.1 Canonical pyroptosis pathway

**Figure 1:**
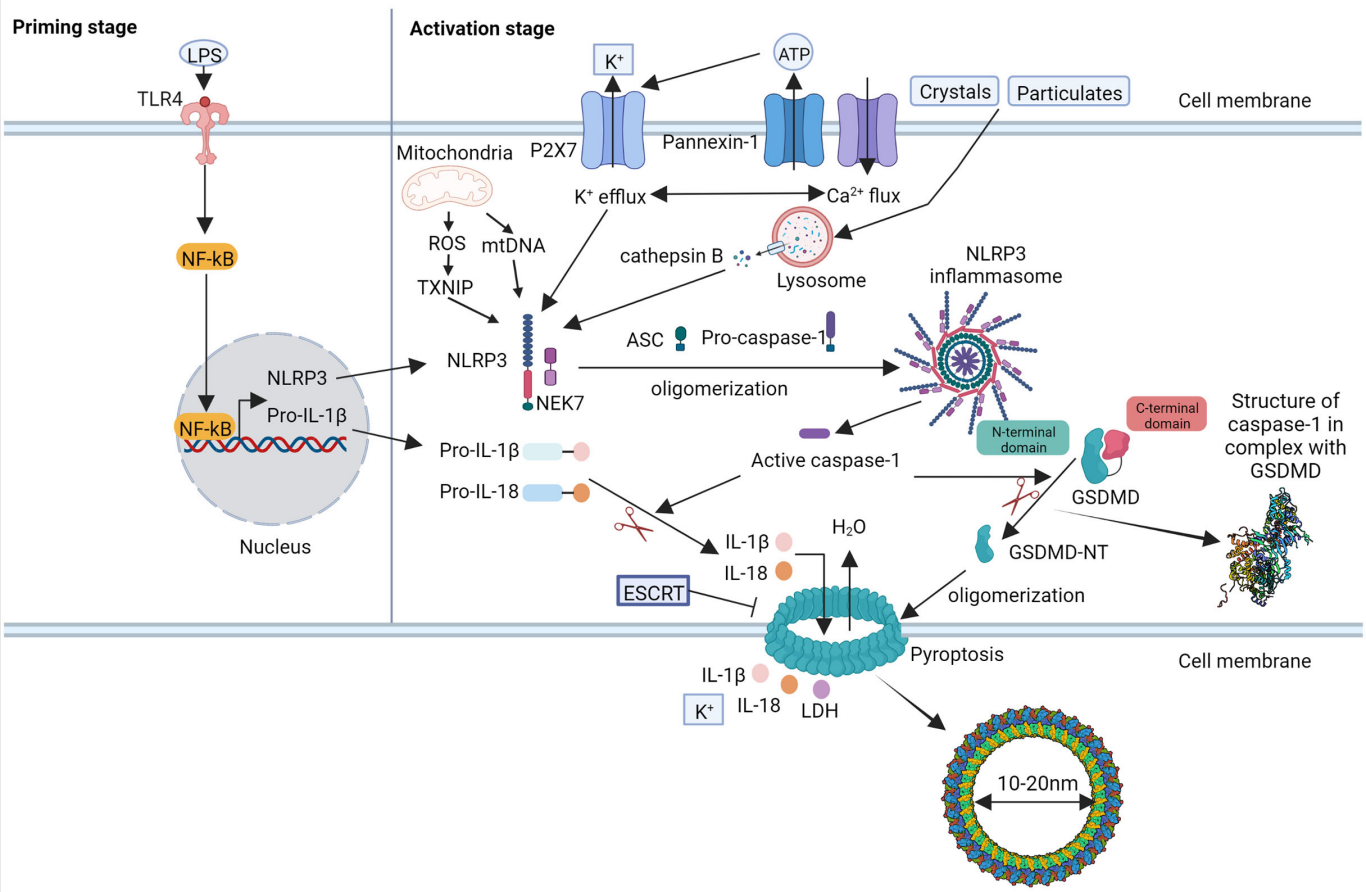
The molecular mechanism of canonical pyroptosis pathway mediated by NLRP3 inflammasome.

Different inflammasomes varies in their composition and the stimuli they can recognize (Sharma and Kanneganti, 2016; Xue et al., 2019). The most common inflammasome in the canonical pathway is the NLR family pyrin domain containing 3 (NLRP3) inflammasome, which is activated by recognizing multiple pathogen-associated molecular patterns (PAMPs) and damage-associated molecular patterns (DAMPs) (**Figure 1**) (Zhao and Zhao, 2020).

The NLRP3 inflammasome comprises three components: the sensor protein NLRP3, apoptosis-associated speck-like protein containing a CARD (ASC), and pre-caspase-1 (Lechtenberg et al., 2014; Sharma and Kanneganti, 2016). There are two steps required for the activation of NLRP3 (Vanaja et al., 2015). Under the stimulation of microbial molecules (such as toll-like receptor [TLR] ligands) or endogenous factors (such as tumor necrosis factor-α), the transcription of pyroptosis-related molecules, such as NLRP3 and pro-interleukin [IL]-1β, increases to a level where NLRP3 activation may occur, which is dependent on the activation of nuclear factor-kappa-gene binding (NF-κB). The second step is the activation and assembly of the NLRP3 inflammasome. Activated by various PAMPs and DAMPs, NLRP3 binds to ASC and recruits the caspase-1 precursor to form the NLRP3 inflammasome. The caspase-1 precursor is cleaved to form the active caspase-1 protein, which subsequently induces pyroptosis. Active caspase-1 cleaves the GSDMD protein into active GSDMD to form pores with an inner diameter of approximately 15 nm on the cell membrane. Active caspase-1 further cleaves precursors IL-1β and IL-18 to form mature IL-1β and IL-18, which are released through the pores and promote inflammation (Ruan et al., 2018; Xia et al., 2021). The pores formed by GSDMD allow ions to enter the cells, which can cause changes in osmotic pressure, cell swelling, and cell rupture accompanied by the release of cellular contents, followed by pyroptosis (Liu et al., 2016). However, the exact molecular mechanisms underlying NLRP3 inflammasome activation remain unclear. Several molecular mechanisms have been proposed, including potassium (K^+^) efflux, mitochondrial dysfunction, release of reactive oxygen species (ROS) and mitochondrial DNA (mtDNA), lysosome disruption, chloride (Cl^−^) efflux, and calcium (Ca^2+^) flux (Lima et al., 2013; Gong et al., 2018; Swanson et al., 2019). Notably, thioredoxin-interacting protein (TXNIP), NIMA-related kinase 7 (NEK7), pannexin-1, and the P2X7 receptor (P2X7R) have recently been shown to be essential for NLRP3 activation. TXNIP is a thioredoxin (TRX)-binding protein that has been shown to participate in several biological processes such as oxidative stress, cell proliferation, and cell apoptosis by inhibiting the function of the TRX system. Under the stimulation of ROS, TXNIP binds to NLRP3 and activates the formation of the NLRP3 inflammasome (Zhou et al., 2010). NEK7, an essential protein downstream of K^+^ efflux, mediates the assembly and activation of the NLRP3 inflammasome (Sharif et al., 2019). Pannexin-1 and P2X7R are associated with K^+^ efflux and changes in ATP levels (Kelley et al., 2019). Notably, it was recently reported that the endosomal sorting complex required for transport (ESCRT)-dependent membrane repair process could remove the GSDMD pores and inhibit pyroptosis (Ruhl et al., 2018). Besides, several studies have discovered that GSDMD pores could regulate IL-1β secretion from living cells without causing cell death, which is called cell hyperactivated state (Evavold et al., 2018; Semino et al., 2018). These results demonstrated that pyroptosis could be regulated, providing basis for targeting pyroptosis in clinical therapy.

### 2.2 Non-canonical pyroptosis pathway

**Figure 2:**
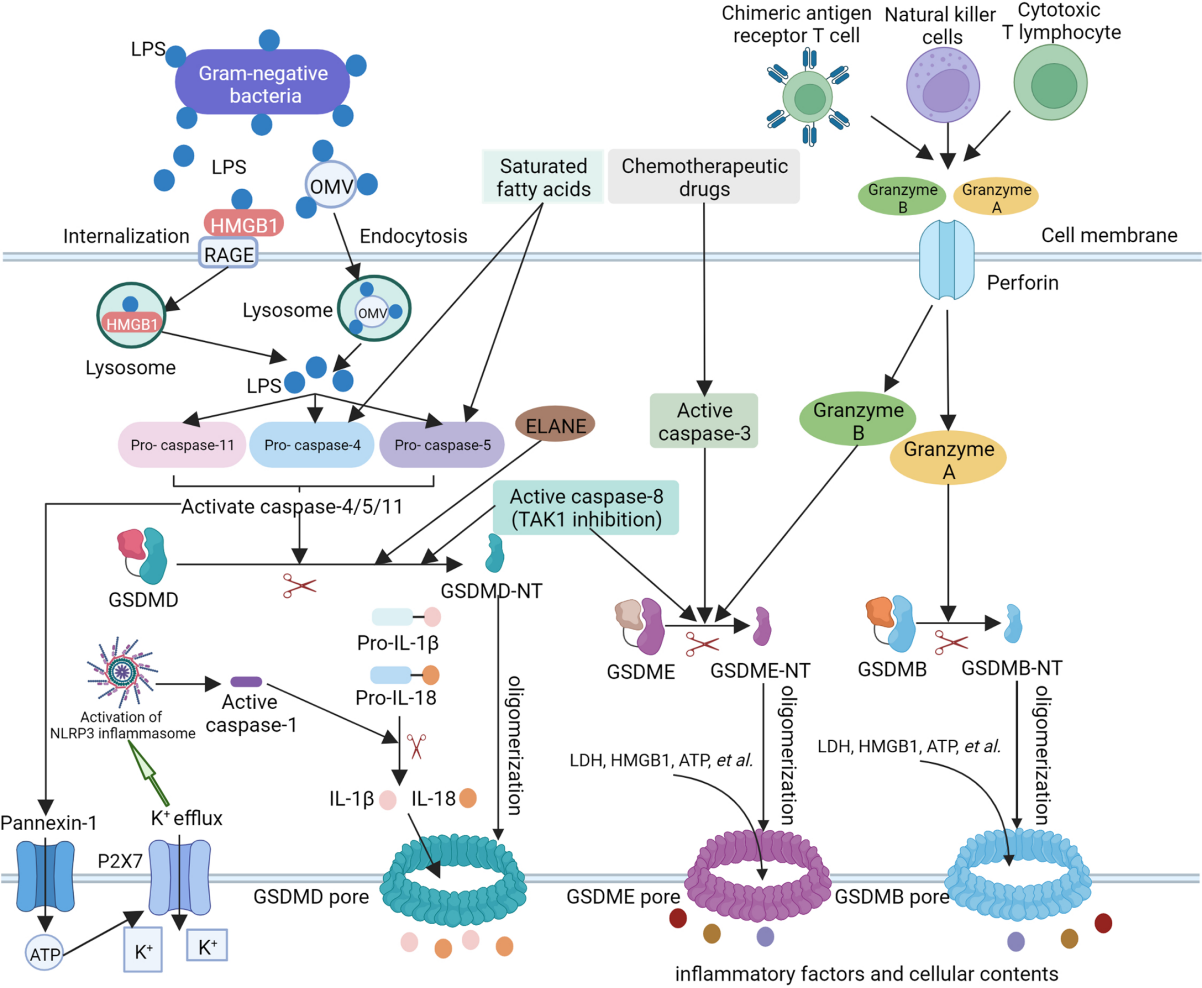
The molecular mechanism of non-canonical pyroptosis pathway and other pyroptosis pathways.

In the non-canonical pyroptosis pathway, caspase-4/5 (human) and caspase-11 (murine) precursors are apical activators. They directly recognize lipid A of lipopolysaccharide (LPS) in the cytoplasm through the caspase activation and recruitment domain (CARD) and form an inflammasome, which leads to their activation. Active caspase-4/5/11 cleave GSDMD to cause pyroptosis (Hagar et al., 2013; Shi et al., 2014; Aglietti et al., 2016; Yi, 2017). Unlike caspase-1, caspase-11 cannot directly cleave pro-IL-1β or pro-IL-18. Instead, caspase-11 indirectly induces the maturation and release of IL-1β and IL-18 by activating the NLRP3/ASC/caspase-1 pathway (Ding and Shao, 2017). The underlying mechanism may be related to the K^+^ efflux induced by pannexin-1 cleaved by caspase-11 (Kayagaki et al., 2011; Ruhl and Broz, 2015; Yang et al., 2015). Additionally, caspase-4-activated GSDMD could form pores on the mitochondrial membrane and generate ROS to activate the NLRP3 inflammasome. These results suggest that there are connections between the non-canonical and canonical pathways (**Figure 2**).

Previous studies have shown that in the non-canonical pyroptosis pathway, gram-negative bacteria can activate the TLR4-TIR-domain-containing adapter-inducing-interferon-β (TRIF) signaling pathway, which results in the production of type I interferons and upregulation of guanylate-binding proteins (GBPs) and immune-related guanosine triphosphatase (GTPase) B10 (IRGB10). Notably, IRGB10 can target outer membrane vesicles (OMVs) carrying LPS, thereby promoting LPS release and entry into the cytoplasm (Vanaja et al., 2016; Santos et al., 2018). In addition, LPS has also been found to enter the cytoplasm by combining with high-mobility group box 1 (HMGB1) (Deng et al., 2018). Several studies have found that in addition to LPS, lipid peroxidation induced by saturated fatty acids can also induce pyroptosis by activating caspase-4/5 (Pillon et al., 2016).

### 2.3 Other pyroptosis pathways

In addition to inflammatory caspases, apoptotic caspases can also cleave the gasdermins family to mediate pyroptosis. For example, unlike GSDMD, GSDME cannot be cleaved by inflammatory caspase-1/4/5/11. However, under the induction of chemotherapeutic drugs, the caspase-3 protein can cleave gasdermin E (GSDME), produce GSDME-NT with a similar effect to that of GSDMD-NT, and induce pyroptosis (Wang et al., 2017; Wang et al., 2018). A study by Orning et al. further found that the activation of caspase-8 during transforming growth factor (TGF)-β-activated kinase 1 (TAK1) inhibition led to the cleavage of GSDMD and GSDME in macrophages, which resulted in pyroptosis (Orning et al., 2018; Sarhan et al., 2018). Additionally, Mandal et al. found that the caspase-8 protein can interact with the caspase-11 protein to amplify inflammatory effects and tissue damage, indicating that caspase-8 participates in pyroptosis (Mandal et al., 2018). Notably, several studies have found that there are caspase-independent gasdermins activation and pyroptosis in recent years. For example, granzyme A (GzmA) and B (GzmB) have been reported to cleave gasdermin B (GSDMB) and GSDME, respectively (Liu et al., 2020; Zhang et al., 2020b; Zhou et al., 2020). In addition, neutrophil elastase (ELANE) was reported to cleave GSDMD and thus produced a fully active NT fragment, though the cleavage site was different from that of caspase-mediated. In turn, GSDMD-NT caused protease activation and nuclear expansion in a feed-forward loop (Kambara et al., 2018; Sollberger et al., 2018). These findings provide new insights into pyroptosis (**Figure 2**).

## 3. The role of pyroptosis in sepsis and sepsis-associated organ dysfunction

### 3.1 The role of pyroptosis in sepsis

Sepsis is a life-threatening organ dysfunction caused by a dysregulated host response to infection, which seriously threatens life and health because of its high morbidity, mortality, and heavy disease burden. The regulation of inflammatory and immune responses contributes to the occurrence and development of sepsis (Barriere and Lowry, 1995; van der Poll and Lowry, 1995; Hotchkiss and Karl, 2003). The host response to infection is initiated when innate immune cells, particularly macrophages, recognize and bind to microbial components. Then, multiple pro-inflammatory and anti-inflammatory mediators are released by the activated macrophages. If the mediators balance each other and the initial infectious insult are overcome, homeostasis will be restored and tissue will be repaired and healed. In contrast, sepsis occurs when the release of pro-inflammatory mediators in response to an infection exceeds the boundaries of the local environment, leading to a more generalized response. The cause may include the effect of the invading microorganisms and the susceptibleness of individuals. When the immune response becomes generalized, widespread cellular injury may occur. The cellular injury, accompanied by the release of pro-inflammatory and anti-inflammatory mediators, often progresses to organ dysfunction. In addition, excess inflammation of sepsis may be followed by immunosuppression, which may aggravate sepsis and sepsis-associated organ dysfunction (Boomer et al., 2011).

Recently, numerous studies have shown that both canonical and non-canonical pyroptosis are involved in the process of sepsis. As a form of pro-inflammatory programmed cell death, pyroptosis functions as a “double-edged sword” in sepsis. A large number of studies have found that pyroptosis can result in exacerbated sepsis. For instance, Li et al. found that the Chinese medicine Babaodan (BBD) can improve the survival of septic mice by diminishing inflammatory cytokine production and improving multiple organ injury. Further experiments have demonstrated that BBD can inhibit both the NF-κB pathway and assembly of the NLRP3 complex in peritoneal macrophages, suggesting that suppressing pyroptosis may be helpful in treating sepsis (Li et al., 2022). In addition, Chen et al. reported that drug-free tea polyphenol nanoparticles (TPNs) exhibited excellent therapeutic efficacy in septic mouse models by scavenging reactive oxygen and nitrogen species and blocking pyroptosis. At the molecular level, TPNs can suppress the pore-forming function of GSDMD by blocking its oligomerization, thus inhibiting pyroptosis (Chen et al., 2022). Furthermore, Wang et al. identified an 8-hydroxyquinoline derivative named 7-[phenyl (pyridin-2-ylamino) methyl] quinolin-8-ol (8-ol, NSC84094), which specifically inhibits HMGB1-mediated caspase-11 signaling. *In vivo* experiments have shown that 8-ol protects mice from sepsis, suggesting that inhibiting caspase-11-dependent non-canonical pyroptosis is also effective in the treatment of sepsis (Wang et al., 2021b). However, several studies have shown that the activation of pyroptosis exerts a protective role in sepsis, especially in the early stage. For example, a prospective study carried out by Garnacho-Montero et al., which included 55 patients with sepsis and 11 critically ill non-septic patients, measured the circulating levels of IL-1β and transcriptional expression of NLRP3 at admission and on days 3 and 7. The results showed that the activation of NLRP3 was more intense in patients with sepsis than in non-septic patients, and the NLRP3 expression was significantly higher on day 7 in patients with sepsis who survived. In addition, an increase in caspase-1 protein expression was observed in survivors compared to that in non-survivors on day 0. These results indicate that sustained NLRP3 activation could be protective during the first week of sepsis (Garnacho-Montero et al., 2020). Similarly, Liu et al. demonstrated that the diminished expression of the NLRP3 inflammasome components contributes to the suppression of monocytes and macrophages in patients with septic shock (Liu et al., 2020). In addition, a study conducted by Huet et al. found that the early activation of NLRP3 by hydrogen peroxide induces the timely and efficient activation of the innate immune response and exerts a protective function (Huet et al., 2017).

In conclusion, pyroptosis participates in sepsis and plays various roles at different stages of the illness. Exploring its regulatory factors may therefore provide a basis for pyroptosis-targeted sepsis therapies.

### 3.2 The role of pyroptosis in sepsis-associated organ dysfunction

An increasing number of studies have explored the role and molecular mechanisms of pyroptosis in SAOD (**Supplementary Table 1**). In sepsis-induced myocardial depression (SIMD), which is a devastating complication of sepsis with a mortality rate of greater than 50%, Kalbitz et al. discovered that the NLRP3 and IL-1β levels were greatly increased in the left ventricular cardiomyocytes of septic mouse models induced by cecal ligation and puncture (CLP). Compared with wild-type (WT) mice, NLRP3 ^-^/^-^ mice showed reduced cardiovascular compromise and plasma levels of IL-1β and IL-6, indicating that NLRP3 is involved in SIMD (Kalbitz et al., 2016). Similarly, Busch et al. showed that compared with septic WT mice, septic NLRP3 knockout mice showed improved survival rates and cardiac function. At the molecular level, IL-1β matures through the activation of the NLRP3 inflammasome, which could further cause atrophy, impair contractility and relaxation, and decrease the deformation of cardiomyocytes (Busch et al., 2021). Recently, Wang et al. revealed that monocyte-derived exosomes could harbor the TXNIP-NLRP3 complex and traffic it to resident heart macrophages, subsequently promoting the maturation of IL-1β and IL-18 in macrophages and aggravating cardiovascular inflammation. In addition, they reported that a small molecule named PSSM1443 could decrease the protein levels of active caspase-1, IL-1β, and IL-18 in SIMD mice by disrupting the interaction of TXNIP-NLRP3. This indicated that inhibiting the activation of the NLRP3 inflammasome is useful for SIMD treatment (Wang et al., 2021). Furthermore, Zhang et al. discovered that carbon monoxide releasing molecule-3 (CORM-3) could improve myocardial function in septic mice by inhibiting NLRP3 inflammasome activation in cardiac fibroblasts. Experiments *in vitro* further showed that CORM-3 prevents the interaction between NLRP3 and ASC (Zhang et al., 2017). These results suggest that pyroptosis is a potential therapeutic candidate for clinical sepsis-induced heart dysfunction.

Regarding sepsis-induced lung injury, which is found in 50% of patients with sepsis and results in poor outcomes, Cheng et al. found that inflammatory caspases participate in endothelial pyroptosis, with human caspase-4/5 in human endothelial cells (ECs) and murine caspase-11 in mice ECs *in vivo*. Compared with control mice, caspase-11^-^/^-^ mice showed a reduction in inflammation and lung injury after CLP for 12 h, indicating that caspase-11 is involved in the pathogenesis of sepsis-induced lung injury (Cheng et al., 2017). Numerous studies have shown that pyroptosis in alveolar macrophages also plays an important role in sepsis-induced lung injury. For instance, Liu et al. found that the Gly-Pro-Ala (GPA) peptide can significantly ameliorate lung tissue injuries, pro-inflammatory cytokine release, and inflammatory cell infiltration in CLP-induced mice. Experiments *in vitro* have shown that the GPA peptide can prevent alveolar macrophages from caspase-1-dependent pyroptosis by decreasing ROS levels (Liu et al., 2021). In addition, it was found that the interaction of NLRP3-NEK7 and subsequent NLRP3 inflammasome assembly and activation can be blocked by 4-[2-(1H-indol-3-yl)-1,3-thiazol-4-yl] benzene-1,2-diol (1,2-diol), which is a 4-benzene-indol derivative. Further experiments *in vivo* have shown that 1,2-diol can alleviate LPS-induced acute lung injury, suggesting that inhibiting pyroptosis may be a novel method of treating sepsis-induced lung injuries (Li et al., 2022).

In a rat model of sepsis-induced brain injury, Zhou et al. found that, compared with those of the sham group, the mRNA and protein expression of NLRP3 and caspase-1 and the levels of inflammatory factors were increased in the cortexes of rats in the CLP group. At the molecular level, the cortical p38 mitogen-activated protein kinase (MAPK) and extracellular signal-regulated kinase (ERK) signaling pathways were activated. Moreover, they discovered that recombinant club cell secretory protein-16 (rCC16) could alleviate pathological changes in the cortex and inhibit the p38 MAPK and ERK signaling pathways and pyroptosis. However, the detailed mechanisms by which rCC16 functions remain unclear (Zhou et al., 2019). In addition, Sun et al. found that dexmedetomidine (DEX) could protect against neuronal injury by attenuating astrocyte pyroptosis through the inhibition of the NLRP3 inflammasome pathway (Sun et al., 2019). Intriguingly, Zhao et al. reported that NLRP3-IL-1β-IL-1R1 signaling is involved in the transition from acute to chronic neuroinflammation in sepsis, which is associated with progressive neurodegeneration and not the initiation of acute neuroinflammation (Zhao et al., 2020).

Pyroptosis is also involved in sepsis-induced kidney injury (SAKI). For example, Li et al. found that the levels of pyroptosis-related proteins were upregulated in the kidney tissues of CLP mice and in LPS-treated human kidney-2 (HK-2) cells. They also discovered that macrophage migration inhibitory factor (MIF) aggravated NLRP3-mediated pyroptosis by promoting the phosphorylation of p65 *in vivo* and *in vitro* (Li et al., 2022). Similarly, Yao et al. reported that DEX could alleviate LPS-induced acute kidney injury by inhibiting NLRP3 inflammasome activation through the suppression of the TLR4/NOX4/NF-κB pathway (Yao et al., 2019).

In sepsis-induced liver injury, Li et al. found that peroxisome proliferator-activated receptor (PPAR)-γ could exert a protective role by inhibiting hepatocyte pyroptosis through the inhibition of the ROS/TXNIP/NLRP3 pathway (Li et al., 2022). Intriguingly, Pai et al. discovered that the prophylactic administration of glutamine (GLN), an abundant free amino acid in the body, could initially upregulate liver pyroptosis to eradicate pathogens 24 h after CLP yet suppress pyroptosis 72 h after CLP. The finding suggested that GLN may regulate the balance of liver pyroptosis at different stages of sepsis, which may provide a basis for pyroptosis-targeted sepsis-induced liver injury treatments (Pai et al., 2020).

Pyroptosis also participates in other SAODs, including intestinal barrier dysfunction, vascular endothelial dysfunction, and disseminated intravascular coagulation (Chen et al., 2019; Chen et al., 2021b; Yuan et al., 2021). All of these studies have demonstrated that pyroptosis plays a critical role in SAOD, indicating that pyroptosis may be a potential target for treatments.

## 4. Regulation of pyroptosis under sepsis

**Figure 3:**
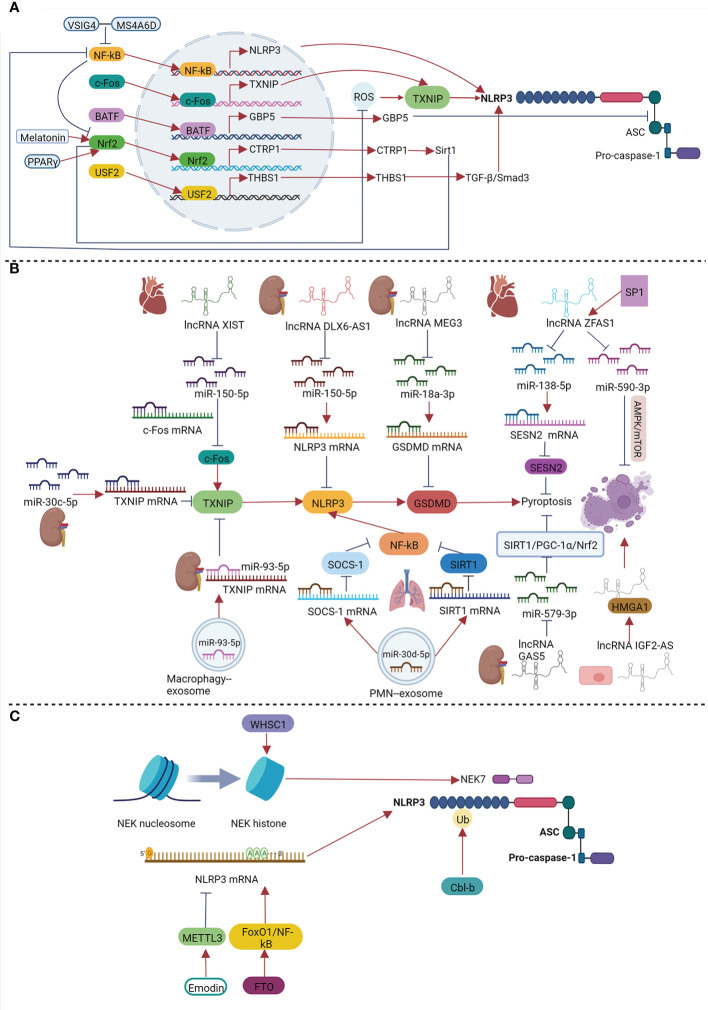
Regulation of pyroptosis under sepsis by transcription factors **(A)**, non-coding RNAs **(B)**, epigenetic modifications and PTMs **(C)**.

The role of pyroptosis in sepsis is related to its severity and stage. Therefore, it is of great significance to explore the factors regulating pyroptosis, which may provide a basis for targeting pyroptosis during sepsis to help in immune protection without causing excessive damage to tissues and organs. Previous studies have shown that under septic conditions, transcription factors, non-coding RNAs (ncRNAs), epigenetic modifications, and post-translational modifications (PTMs) can play vital roles in regulating pyroptosis (**Figure 3**).

### 4.1 Regulation of pyroptosis by transcription factors

Transcription factors are a class of proteins that can bind to specific sequences upstream of the 5’ end of genes to regulate their transcription. Various transcription factors participate in the regulation of pyroptosis in sepsis. Transcription factors can function by directly regulating the transcription of genes encoding proteins involved in pyroptosis-related pathways. For example, NF-κB is an important transcription factor that plays key roles in a variety of biological processes, such as inflammation, immunity, and cell death. As previously mentioned, NF-κB plays a role in the priming step of NLRP3 inflammasome activation, which ensures the activation and assembly of the NLRP3 inflammasome by increasing the transcriptional expression of NLRP3 and pro-IL-1β. Chen et al. found that the inhibition of p65 could significantly inhibit the decrease in cell viability in LPS-treated brain microvascular endothelial cells (BMECs) and reduce the expression of GSDMD. They also reported that p65 could directly bind to the *NLRP3* promoter, indicating that p65 can directly regulate the expression of NLRP3 in LPS-induced BMECs (Chen et al., 2020). In addition, V-set and immunoglobulin domain containing 4 (VSIG4), a complement receptor of the immunoglobulin superfamily, was also found to inhibit the transcription of NLRP3 and IL-1β in macrophages *in vitro* and *in vivo*. At the molecular level, the VSIG4 protein could bind to member 6D of the membrane spanning 4-domains subfamily A (MS4A6D) and form an inhibitory signaling complex, which inhibited NF-κB (Huang et al., 2019; Shi et al., 2020). This study illustrated the essential role of NF-κB in NLRP3 inflammasome-mediated pyroptosis. Considering that NF-κB is a canonical transcription factor involved in inflammasomes and oxidative stress, we speculate that NF-κB can also modulate the second stage of NLRP3 inflammasome activation by promoting inflammation and ROS.

Transcription factors can also affect the activation and assembly of inflammasomes. The c-Fos protein, which is encoded by the *Fosl2* gene, can form a transcription factor complex activator protein 1 (AP-1) with c-Jun in the nucleus. AP-1 participates in various biological processes, including inflammation and apoptosis. For example, Wang et al. found that the c-Fos protein can bind to the promoter region of *TXNIP*, thereby promoting pyroptosis in LPS-induced H9c2 cardiomyocytes (Wang et al., 2021a). Moreover, basic leucine zipper transcription factor ATF-like protein (BATF), a member of the AP-1 transcription factor family, participates in the regulation of the immune response (Murphy et al., 2013). Guo et al. further found that the BATF protein can promote the activation of the NLRP3 inflammasome by enhancing the transcription of guanylate-binding protein 5 (GBP5) in sepsis-induced liver injury (SALI), thus aggravating liver injury (Guo et al., 2021). GBP5 is a member of the GTPase superfamily, which has its own effect on host innate and cell-autonomous immunity (Praefcke, 2018). A previous study showed that GBP5 can promote NLRP3 inflammasome-mediated pyroptosis by facilitating the formation of the NLRP3-ASC complex (Shenoy et al., 2012).

Other transcription factors mediate pyroptosis by regulating stimulating factors or molecules (e.g., ROS). Nuclear factor erythroid 2 related factor (Nrf2) is a canonical transcription factor that regulates cellular oxidative stress responses and participates in maintaining redox homeostasis. In recent years, numerous studies have shown that Nrf2 can inhibit pyroptosis and play a regulatory role in a variety of diseases, including sepsis (Rahim et al., 2021; Li et al., 2022). Li et al. found that PPAR-γ could inhibit the ROS/TXNIP/NLRP3 signaling pathway by activating *Nrf2* gene expression to inhibit hepatocyte pyroptosis, which ultimately had a protective effect against sepsis-induced liver injury (Li et al., 2022). Similarly, Rahim et al. revealed that melatonin can ameliorate sepsis-induced myocardial injury by activating the Nrf2-related pathway and inhibiting the formation of the NLRP3 inflammasome (Rahim et al., 2021). Teng et al. found that, in addition to inhibiting ROS, Nrf2 could have a protective role by upregulating C1q/tumor necrosis factor-related protein 1 (CTRP1) expression and inhibiting pyroptosis by binding to the promoter of *CTRP1* in sepsis-induced myocardial injury *in vivo* and *in vitro* (Teng et al., 2022). CTRP1 is a homologue of adiponectin. Previous studies have shown that the overexpression of CTRP1 can improve the survival rate and heart function in LPS-treated mice by inhibiting myocardial inflammation, oxidative damage, and cellular apoptosis. The underlying molecular mechanism involves CTRP1 protein-activated sirtuin 1 (*SIRT1*) gene expression and the inhibition of the NF-κB pathway to promote *Nrf2* gene expression. Based on these two studies, it is speculated that the transcription factors Nrf2 and CTRP may inhibit oxidative damage in sepsis-induced myocardial injury through positive feedback and play an important protective role. In addition to ROS, transcription factors can affect pyroptosis by regulating its associated molecules. For example, upstream stimulatory factor 2 (USF2), which is a member of the basic helix-loop-helix-leucine zipper transcription factor family, can regulate a variety of cellular processes (Sirito et al., 1992; Chi et al., 2020). In SAKI, Sun et al. found that the knockdown of USF2 led to the inhibition of the TGF-β/suppressor of mothers against decapentaplegic (Smad) 3 signaling pathway by downregulating the expression of thrombospondin1 (THBS1), thereby reducing pyroptosis and improving renal injury (Sun et al., 2022). TGF-β belongs to the TGF-β superfamily, and previous studies have shown that it is involved in the activation of the NLRP3 inflammasome (Zhang et al., 2020).

Transcription factors can therefore regulate pyroptosis in sepsis, especially SAOD, including its stimulation signals and the priming, activation, and assembly of the NLRP3 inflammasome.

### 4.2 Regulation of pyroptosis by non-coding RNAs

NcRNAs are a series of RNAs that do not encode proteins, including long non-coding RNAs (lncRNAs), microRNAs (miRNAs), and circular RNAs (circRNAs). They participate in and regulate various biological processes. Recent studies have shown that ncRNAs can be involved in the occurrence and development of various diseases, including sepsis, by regulating pyroptosis.

#### 4.2.1 The role of long non-coding RNAs in pyroptosis in sepsis

LncRNAs are a class of ncRNAs that are more than 200 nucleotides in length. Related studies have suggested that lncRNAs can bind to DNA, RNA, and proteins, thus inhibiting or activating gene expression through complex biological mechanisms (Borkiewicz et al., 2021). Several studies have shown that the regulation of pyroptosis by lncRNAs is important in sepsis.

The lncRNA zinc finger antisense 1 (ZFAS1) has regulatory roles in various tumors. In 2021, An *et al.* found that lncRNA ZFAS1 expression was downregulated in the sera of patients with SIMD compared to that in healthy individuals. The overexpression of lncRNA ZFAS1 *in vitro* ameliorated the decrease in cell viability and increase in the levels of pyroptosis-associated proteins and inflammatory factors in LPS-induced H9c2 cardiomyocytes, indicating that the upregulation of lncRNA ZFAS1 expression could alleviate LPS-induced cardiomyocyte pyroptosis. Mechanistically, lncRNA ZFAS1 acts by upregulating sestrin2 (SESN2) expression through the inhibition of miR-138-5p by directly binding to it. In addition, a previous study using an LPS-induced septic rat model found that myocardial tissue damage and inflammatory cell infiltration were reduced following the overexpression of lncRNA ZFAS1, which further indicated that lncRNA ZFAS1 had a protective effect and could improve myocardial injury by inhibiting LPS-induced cardiomyocyte pyroptosis (An et al., 2021). Notably, Liu *et al.* came to the opposite conclusion based on a CLP-induced septic mouse model and LPS-treated mouse primary cardiomyocytes (Liu et al., 2020). They found that the expression of lncRNA ZFAS1 was upregulated in the myocardial tissues of mice in the CLP group compared with that in the control. However, inhibiting the expression of ZFAS1 could improve cardiac function, suggesting that ZFAS1 aggravates myocardial injuries in sepsis. Mechanistically, experiments *in vitro* showed that the upregulation in lncRNA ZFAS1 expression was caused by the activation of its transcription by the transcription factor specificity protein 1. LncRNA ZFAS1 could further act as a competing endogenous RNA (ceRNA) to inhibit the AMP-activated protein kinase (AMPK)-mammalian target of rapamycin (mTOR) pathway by suppressing miR-590-3p, thereby aggravating SIMD by inhibiting autophagy and promoting pyroptosis (Liu et al., 2020). The reason for this difference may be that the expression of lncRNA ZFAS1 changes dynamically at different stages of sepsis. Notably, there are differences between the circulatory manifestations of LPS-induced and CLP-induced septic models, as the former is hypodynamic. Thus, the expression of lncRNA ZFAS1 may be different in the heart tissues of the models. Various miRNAs may be downstream of lncRNA ZFAS1; therefore, it may also participate in other cellular processes in addition to pyroptosis. In future studies, it will be of great significance to dynamically monitor the changes in lncRNA ZFAS1 in SIMD and to explore the main downstream pathways and cellular processes regulated by lncRNA ZFAS1 at different periods. In addition to lncRNA ZFAS1, lncRNA X-inactive specific transcript (XIST) also participates in the regulatory process of pyroptosis in SIMD. For example, Wang *et al.* found that the expression of lncRNA XIST was upregulated *in vitro* and *in vivo*, and XIST acts as a ceRNA to inhibit miR-150-5p and promote the expression of the *c-Fos* gene. On one hand, c-Fos bound to the promoter of the *TXNIP* gene to promote TXNIP-mediated pyroptosis; on the other hand, it bound to the promoter of the *XIST* gene to upregulate the expression of XIST, which promoted the XIST/miR-150-5p/c-Fos axis through positive feedback. Moreover, experiments *in vivo* showed that after inhibiting XIST, the expression of pyroptosis-associated proteins in myocardial tissue was downregulated, and myocardial injury was alleviated (Wang et al., 2021a). These results provide new insights for the treatment of SIMD.

The regulation of pyroptosis by lncRNAs can also influence the development of SAKI. In a mouse SAKI model established using CLP and HK-2 cells induced by LPS, Ling *et al*. found that lncRNA growth arrest specific 5 (GAS5) could activate the SIRT1/PPAR-γ coactivator-1α/Nrf2 signaling pathway by targeting miR-579-3p and inhibiting pyroptosis. The overexpression of lncRNA GAS5 or knockdown of miR-579-3p could enhance the expression of the *SIRT1* gene, improve the survival rate of mice, and relieve kidney injuries (Ling et al., 2021). In addition, the levels of lncRNAs maternally expressed 3 and distal-less homeobox 6 antisense RNA 1 were also reported to be elevated in SAKI and could promote pyroptosis in renal tubular epithelial cells induced by LPS by regulating the miR-18a-3p/GSDMD and miR-223-3p/NLRP3 signaling pathways, respectively. However, these studies lacked *in vivo* experiments to verify whether the inhibition of these two pathways had a protective effect in SAKI (Tan et al., 2020; Deng et al., 2021).

Additionally, pyroptosis regulated by lncRNAs also participates in the repair of vascular endothelial injury in sepsis. Liang *et al.* extracted and identified endothelial progenitor cells (EPCs) from the peripheral blood collected from healthy individuals and patients with sepsis. The results showed that the lncRNA insulin-like growth factor 2 antisense RNA (IGF2-AS) was highly expressed in EPCs from the septic group compared with the healthy group. Further studies showed that lncRNA IGF2-AS could bind to the high mobility group protein A1 to regulate nucleotide metabolism and promote the pyroptosis of EPCs. This study also preliminarily explored the effect of exosomal lncRNA IGF2-AS from mesenchymal stem cells (MSCs) in sepsis. The results showed that the MSC-derived exosomal lncRNA IGF2-AS could promote the pyroptosis of EPCs in patients with sepsis, providing insights for potential therapeutic targets for sepsis (Liang et al., 2022).

#### 4.2.2 The role of miRNAs in pyroptosis in sepsis

As we know, miRNAs are a class of ncRNAs that are approximately 22–26 nucleotides in length. They regulate target gene expression at the post-transcriptional level by binding to the 3’-untranslated region (UTR) of downstream genes (Huang, 2017; Subramanian and Steer, 2019). Numerous studies have found that miRNAs participate in sepsis and SAODs (Ho et al., 2016; Ghafouri-Fard et al., 2021). For example, Sun *et al.* found that exosomal miR-27b derived from MSCs could inhibit sepsis in CLP-induced mice and attenuate inflammation in LPS-mediated bone marrow-derived macrophages (BMDMs). The underlying molecular mechanism was that miR-27b could inhibit JMJD3 and inactivate the NF-κB signaling pathway (Sun et al., 2021). In addition, Chen *et al.* reported that microRNA-23a-5p aggravated LPS-induced acute lung injury by targeting HSP20/ASK1 (Chen et al., 2021). These results suggested that miRNAs were involved in sepsis and SAODs.

Recently, miRNAs have been found to be invloved in sepsis by regulating pyroptosis (Li et al., 2021). In addition to miRNAs, which play a regulatory role in pyroptosis in sepsis as downstream lncRNAs, Li *et al*. revealed that miR-30c-5p expression was significantly downregulated in the kidney tissues of septic mice and HK-2 cells induced by LPS compared with control groups. They discovered that the overexpression of miR-30c-5p using a miR-30c-5p mimic could lead to the downregulation of the expression of NLRP3, caspase-1, and ASC as well as downstream cytokines such as IL-1β and IL-18 in HK-2 cells induced by LPS and ATP. This indicated that miR-30c-5p is involved in the process of SAKI through the regulation of NLRP3/caspase-1-mediated pyroptosis. Further experiments showed that the *TXNIP* gene was the target of miR-30c-5p, and miR-30c-5p inhibited pyroptosis by inhibiting *TXNIP* gene expression. Moreover, the mice that were administered kidney-specific injections of the miR-30c-5p mimic showed decreased tubular cavity expansion, vacuolar degeneration of tubular epithelial cells, and expression of inflammatory factors compared with the control group. In addition, the protein expression of TXNIP, NLRP3, caspase-1, and ASC in renal tissues was inhibited. Similarly, the kidney-specific knockdown of TXNIP protected against renal injury and significantly downregulated the expression of pyroptosis-associated proteins, further indicating that miR-30c-5p could negatively regulate NLRP3-mediated pyroptosis by inhibiting *TXNIP* gene expression (Li et al., 2021). Considering that the molecular mechanisms of miRNAs are relatively definite and miRNAs can not only regulate pyroptosis-associated proteins directly but also mediate the regulation of lncRNAs, miRNAs may be a valuable research topic for sepsis and SAOD.

#### 4.2.3 The role of exosome-driven non-coding RNAs in pyroptosis in sepsis

Exosomes are extracellular vesicles secreted by various cells. They can transfer proteins and genetic material (including DNA, mRNA, and miRNA) to target cells and play a profound role in intercellular communication. In addition to the MSC-derived exosomal lncRNA IGF2-AS (Liang et al., 2022), the ncRNAs delivered by exosomes may have crucial roles in sepsis. For example, Jiao *et al.* found that exosomal miR-30d-5p of neutrophils (polymorphonuclear neutrophils) could activate macrophage pyroptosis and aggravate lung injury in sepsis-induced acute lung injury by activating NF-κB. The molecular mechanisms involved exosomal miR-30d-5p targeting suppressor of cytokine signaling 1 and SIRT1 genes in macrophages and subsequently activating NF-κB in part by increasing the acetylation of lysine 310 in p65 (Jiao et al., 2021). In addition, Juan *et al.* found that M1 macrophages aggravated the level of pyroptosis in the LPS-induced mouse kidney epithelial cell line TCMK-1, whereas M2 exerted the opposite effect. Further experiments *in vitro* and *in vivo* revealed that this opposite effect was caused by the difference in the expression of miR-93-5p carried by M1 and M2. The expression of miR-93-5p was significantly upregulated in M2 exosomes, and it could directly target TXNIP to inhibit the pyroptosis of renal tubular epithelial cells (Juan et al., 2021). In summary, the intercellular interactions mediated by exosome-derived ncRNAs may contribute to novel strategies for the treatment of sepsis.

In conclusion, the regulation of pyroptosis by ncRNAs plays a vital role in sepsis and may be a potential therapeutic target. However, there are still many gaps in this area, such as the lack of studies on circRNAs and exploring the potential roles of circRNAs and ncRNAs in other organ injuries during sepsis. These studies include those on sepsis-induced liver and brain injuries and the ncRNA regulation of caspase-1/4/5/11 and related clinical studies.

### 4.3 Regulation of pyroptosis by epigenetic modifications in sepsis

Epigenetic modifications are heritable changes in gene expression that do not change the nucleotide sequence of the gene. They include DNA and histone modifications. m6A methylation, which has attracted much attention in recent years, is an epigenetic modification. Studies have shown that various epigenetic modifications are closely related to the activation or inhibition of pyroptosis and ultimately affect pyroptosis-related diseases, including sepsis. For instance, Wang *et al*. found that emodin could upregulate the expression of methyltransferase-like 3 (METTL3) in sepsis-induced brain injury *in vitro*, thereby downregulating the mRNA and protein expression of NLRP3 and ultimately inhibiting pyroptosis and inflammation (Wang et al., 2022). In addition, m6A demethylase fat mass and obesity-associated proteins (FTOs) has also been found to regulate pyroptosis in sepsis. Luo *et al*. found that in a septic shock mouse models induced by LPS, FTO inhibition suppressed the NLRP3 inflammasome formation by inhibiting the FoxO1/NF-κB signaling pathway, thus reducing tissue damage and improving survival (Luo et al., 2021). Moreover, Liu *et al.* discovered that the level of the SET domain-containing histone methyltransferase Wolf-Hirschhorn syndrome candidate gene 1 (WHSC1) was elevated in LPS-induced septic lung injury, and WHSC1 knockout attenuated LPS-induced lung injury and alveolar macrophage pyroptosis. Further mechanistic studies have revealed that WHSC1 exacerbates alveolar macrophage pyroptosis in sepsis-induced ALI through the NEK7-mediated activation of the NLRP3 inflammasome (Liu et al., 2021). Previous studies have also shown that WHSC1 can regulate NEK7 *via* lysine dimethylation at position 36 of histone H3 (H3K36me2), thereby deteriorating head and neck squamous cell carcinoma (Saloura et al., 2015). Unfortunately, the detailed mechanisms by which WHSC1 regulates NEK7 expression in sepsis have not been explored and require further study. In conclusion, the modulation of pyroptosis by epigenetic modifications may be a new regulatory mechanism in the process of sepsis. However, in the future, many experimental studies are needed to explore the roles and mechanisms of various epigenetic modifications in regulating the expression of genes related to pyroptosis and the resulting impact on pyroptosis in sepsis.

### 4.4 Regulation of pyroptosis by PTMs in sepsis

PTMs refer to the chemical modifications of proteins after translation, including phosphorylation, acetylation, ubiquitination, glycosylation and succination. These play crucial roles in regulating protein activity. Previous studies have shown that various PTMs of pyroptosis-associated proteins influence NLRP3 inflammasome and pyroptosis (Song and Li, 2018; Kelley et al., 2019). For example, Humphries *et al.* found that dimethyl fumarate(DMF) or endogenous fumarate could modify GSDMD at critical cysteine residues to form S-(2-succinyl)-cysteine, resulting in inactivating GSDMD and inhibiting pyroptosis. In addition, they found that DMF could also succinate GSDME and inhibit GSDME-driven cell death comparably (Humphries et al., 2020). Besides, Mortimer *et al*. showed that the phosphorylation of NLRP3 at Ser295 (mouse NLRP3 Ser291) by protein kinase A (PKA) could inhibit NLRP3 inflammasome activation by inhibiting NLRP3 ATPase activity (Mortimer et al., 2016). However, the roles of PTMs in regulating pyroptosis during sepsis remain poorly understood. In 2020, Tang *et al.* found that the E3 ubiquitin ligase Casitas-B-lineage lymphoma protein-b (Cbl-b) was essential for preventing endotoxemia induced by a sub-lethal dose of LPS in a caspase-11/NLRP3-dependent manner (Tang et al., 2020). At the molecular level, the NLRP3 protein underwent both K63- and K48-linked polyubiquitination. The Cbl-b protein bound to the K63-ubiquitin chains attached to the NLRP3 leucine-rich repeat domain (LRR) *via* its ubiquitin-associated region (UBA) and targeted NLRP3 at K496 for K48-linked ubiquitination and proteasome-mediated degradation. The study also identified ring finger protein 125 as an additional E3 ubiquitin ligase that initiated the K63-linked ubiquitination of the NLRP3 LRR domain. In summary, the NLRP3 protein was sequentially ubiquitinated by K63- and K48-linked ubiquitination, thus preventing the NLRP3 inflammasome activation and restraining endotoxemia. Considering the profound effects of PTMs on pyroptosis-associated proteins, PTMs may be potential strategies to regulate pyroptosis and improve sepsis and should be studied in the future.

In summary, during sepsis, transcription factors, ncRNAs, epigenetic modifications, and PTMs all have regulatory effects on pyroptosis and ultimately result in the regulation of sepsis and SAOD. However, the detailed regulatory mechanisms remain unclear and require further study. In addition, most existing studies have been conducted at the cellular or animal level. Thus, further clinical studies are needed to evaluate the modulation of pyroptosis in patients with sepsis.

## 5. Interaction of pyroptosis with other programmed cell death pathways in sepsis

**Figure 4:**
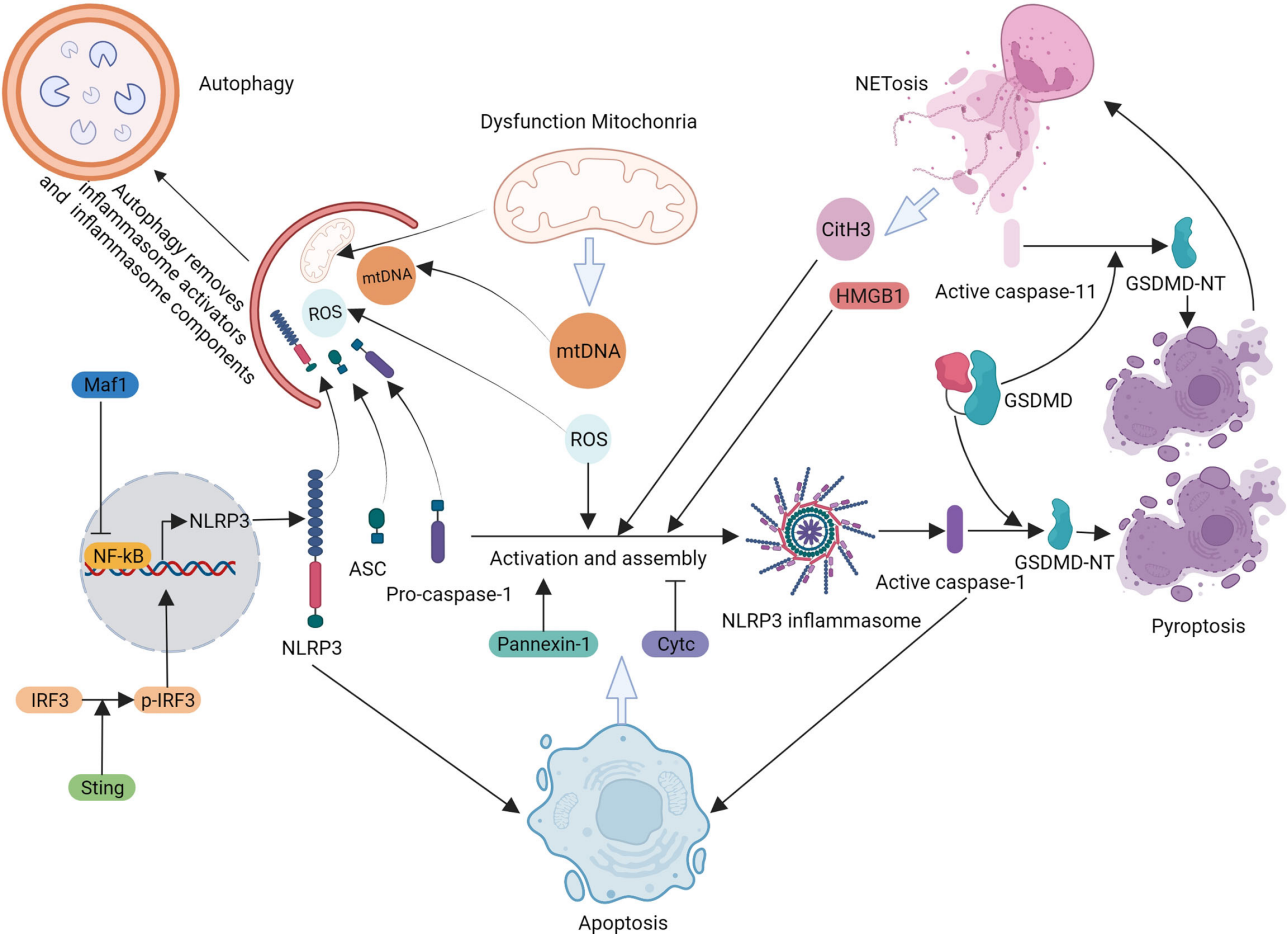
Crosstalk between pyroptosis and other programmed cell deaths under sepsis.

In 2018, the Nomenclature Committee on Cell Death issued the latest guidelines on the classification and nomenclature of cell death, dividing cell death into accidental cell death (ACD) and programmed cell death (PCD) (Galluzzi et al., 2018). ACD refers to the instantaneous and catastrophic death of cells exposed to severe physical, chemical, or mechanical insults. In contrast, PCD relies on dedicated molecular machinery and can occur under physiological or pathological conditions. PCD can be modulated by pharmacological or genetic interventions, which have attracted much attention in recent research on sepsis (**Table 1**). Numerous studies have shown that in addition to pyroptosis, PCD pathways such as autophagy, apoptosis, NETosis, necroptosis, and ferroptosis are involved in sepsis. Notably, pyroptosis and other PCD pathways are interdependent, rather than independent, in sepsis (**Figure 4**).

**Table 1:** Differences among Various Cell Deaths.

	Necrosis (Chen et al., 2018; Galluzzi et al., 2018)	Apoptosis (Strasser et al., 2000; Flusberg and Sorger, 2015)	Pyroptosis (Shi et al., 2014; Xue et al., 2019)	Ferroptosis (Xie et al., 2016; Yang and Stockwell, 2016)	Autophagy (Mizushima and Levine, 2010; Das et al., 2012; Galluzzi et al., 2017)	Necroptosis (Pasparakis and Vandenabeele, 2015; Grootjans et al., 2017)	NETosis (Li et al., 2010; Papayannopoulos et al., 2010; Thiam et al., 2020)
**Programmed cell death**	No	Yes	Yes	Yes	Yes	Yes	Yes
**Proinflammatory**	Yes	No	Yes	Yes	Partially	Yes	Yes
**Key molecules**	–	Casapse-2/3/6/7/8/9/10, Bcl/Bax	Caspase1/4/5/11, Gasermins family	Glutathione peroxidase 4(GPX4), Fe^2+^	Autophagy-related Genes (ATGs), LC3	Receptor interacting protein kinase(RIPK)1, RIPK3, mixed lineagekinase domain like pseudokinase (MLKL)	Neutrophil Elastase(NE), Myeloperoxidase(MPO), Peptidearginine Deaminase 4(PAD4)
**Dependent on caspases**	No	Yes	Yes	No	No	No	No
**Initiation Factors**	Physical, chemical, or mechanical stress	Extrinsic/ intrinsic signals	Activation of inflammas‐ omes by extrinsic/ intrinsic stress	Iron accumulation and lipid peroxidation	Injury, stress	Activation of specific death receptors or PRRs upon caspase-8 inactive	Various microbial and sterile activators or upon stimulation of specific receptors including (but not limited to) TLRs
**Nucleus**	Pyknosis, karyorrhexis, karyolysis	Chromatin condensation , nuclear fragmentation	Chromatin condensation	No chromatin condensation	No chromatin condensation	Rupture	Extrusion of chromatin fibers intermixed with cytoplasmic and nuclear components
**Cytoplasm**	Swelling	Cytoplasm shrinkage	Swelling and then flatting	Mitochondrial shrinkage, an electron-dense ultrastructure, reduced/disappeared cristae, and ruptured outer mitochondrial membrane	Extensive cytoplasmic vacuolization	Swelling, mitochondrial dysfunction	No swelling
**Cell membrane**	Cell membrane ruptures, releasing cell contents	Intanct, membrane blebbing, formation of apoptotic bodies	Membrane pore formation, cell lysis, formation of pyroptotic body	No blebbing	Cell membrane Intact	Membrane pore formation, releasing cell contents	Plasma membrane rupture
**Cell lysis**	Yes	No	Yes	Yes	No	Yes	Yes/No
**Commonly used detection methods**	Electron microscopy, LDH release assay, qRT-PCR, western blot, IF, IP	Electron microscopy, Flow cytometry, TUNEL assay, qRT-PCR, western blot, IP, IF	Electron microscopy, Flow cytometry, TUNEL assay, LDH release assay, qRT-PCR, western blot, Elisa, IP, IF,	Electron microscopy, ROS, Fe^2+^, qRT-PCR, western blot	Electron micros‐ copy, qRT-PCR, western blot, GFP-LC3	Electron microscopy, flow cytometry, TUNEL assay, qRT-PCR, western blot, IP, IF	Flow cytometry, western blot, Elisa, IP, IF

### 5.1 Interaction of pyroptosis and autophagy in sepsis

Autophagy is a conserved intracellular process that leads to the degradation and recycling of damaged cellular proteins and organelles and the destruction of intracellular pathogens (Mizushima and Levine, 2010; Ren et al., 2017). Autophagy contributes to the maintenance of cellular homeostasis and plays an important role in regulating immune and inflammatory responses. In sepsis-related studies, autophagy has been shown to participate in regulating NLRP3 inflammasome activation. For instance, Pu *et al.* reported that autophagy-related protein 7 (Atg7) is involved in inflammasome activation in sepsis induced by *Pseudomonas aeruginosa*. In septic Atg7^fl/fl^ mice, IL-1β levels in the bronchoalveolar lavage fluid (BALF) and blood as well as ASC and IL-18 protein levels in the lungs were significantly elevated compared to those in the septic WT mice. *In vitro*, pyroptosis was increased in macrophages upon *P. aeruginosa* infection, as evidenced by increased caspase-1 and ASC protein levels. These results indicated that Atg7 plays an essential role in the inhibition of inflammasome activation. Mechanistically, flagellin, a conserved PAMP, may be a potential mediator of inflammasome hyperactivation in Atg7-deficient conditions during *P. aeruginosa* infection (Pu et al., 2017). Similarly, compared with WT bone marrow-derived macrophages (BMMs), autophagy-deficient Atg5^-/-^and Atg14^-/-^ BMMs secrete elevated amounts of IL-1β and IL-18 proteins, forming ASC specks more frequently, after LPS treatment (Finethy et al., 2020). In addition, Ge *et al.* found that the inhibition of Beclin1 using Beclin1 small interfering RNA significantly enhanced the protein expression of NLRP3, ASC, caspase-1, and IL-1β in LPS-stimulated macrophages (Ge et al., 2019). Beclin1 is a protein required for the initiation of autophagy. Notably, there are various pathways involved in autophagy. Mitophagy is a selective autophagy that can specifically remove damaged or excess mitochondria and maintain cellular homeostasis (Wang et al., 2021b). In 2016, Kim *et al.* found that SESN2 could suppress sepsis by inducing mitophagy and inhibiting NLRP3 activation in macrophages. The molecular mechanism was that mitophagy could inhibit the excessive generation of mitochondrial ROS and release of mtDNA, which could activate the NLRP3 inflammasome and induce inflammation (Kim et al., 2016). Similarly, Li *et al.* revealed that bone marrow-derived mesenchymal stem cells (BMSCs) could restrict NLRP3 inflammasome activation by increasing mitophagy and decreasing mitochondrial ROS levels in macrophages. In C57BL/6 mice after CLP, BMSC treatment exerted beneficial effects, as evidenced by improved survival rates and multiorgan functions (Li et al., 2018). Liu *et al.* discovered that buformin (BF) could alleviate sepsis-induced acute lung injury by inhibiting NLRP3-mediated pyroptosis *in vivo* and *in vitro*. Further studies have found that BF inhibits pyroptosis in macrophages by enhancing autophagy *via* the AMPK-mTOR pathway. Notably, compared with the LPS + BF treatment, the autophagy inhibitor 3-MA upregulated the protein level of NLRP3, but not its mRNA level, suggesting that the autophagy promoted by BF most likely promoted the degradation of the NLRP3 inflammasome (Liu et al., 2022). In summary, autophagy can inhibit the activation of the NLRP3 inflammasome not only by removing endogenous NLRP3 inflammasome activators, such as ROS and DAMPs, but also by removing NLRP3 inflammasome components (Biasizzo and Kopitar-Jerala, 2020). Thus, the activation of autophagy may be a beneficial strategy for sepsis treatment. For instance, in a septic rat model, Yang *et al.* found that DEX could attenuate renal injury by significantly reducing the expression of NLRP3 inflammasome-mediated pyroptosis pathway proteins NLRP3, ASC, caspase-1, and cleaved caspase-1 and inflammatory factors IL-1β and IL-18. The molecular mechanism was that DEX enhanced autophagy *via* α2-AR/AMPK/mTOR signaling and subsequently inhibited the activation of the NLRP3 inflammasome (Yang et al., 2020). Similarly, in sepsis-induced acute lung injury, the knockdown of geranylgeranyl pyrophosphate synthase large subunit 1 was found to attenuate injury by suppressing NLRP3 inflammasome activity through the promotion of autophagy (Li et al., 2021).

Intriguingly, NLRP3 inflammasome-mediated pyroptosis regulates autophagy during sepsis. For instance, Jin *et al.* reported that similar levels of neutrophil recruitment to the peritoneum but improved survival were observed in NLRP3^-/-^ mice compared with those of WT mice. Considering the critical role of neutrophils in bacterial clearance, it was speculated that there may be a difference in neutrophilic functions between WT and NLRP3^-/-^ mice. Subsequent experiments showed that peritoneal cells (primarily neutrophils) and lung tissues from NLRP3^-/-^ mice 24 h post-CLP displayed decreased autophagy, characterized by a reduction in Atg7 levels, inhibition of microtubule-associated protein light chain 3-II formation, and enhanced sequestosome-1 levels. The experiment also found that phagocytosis was augmented in NLRP3^-/-^ neutrophils, which may have been associated with an increased expression of macrophage receptors with collagenous structures and mannose-binding lectins (Jin et al., 2017).

Taken together, pyroptosis and autophagy can interact and regulate the immune and inflammatory reactions in sepsis. In the future, targeting this relationship could be a possible treatment option for patients with sepsis.

### 5.2 Interaction of pyroptosis and apoptosis in sepsis

Although pyroptosis and apoptosis are both PCD mediated by caspase proteins, they differ in mechanisms, biological characteristics and functions. Pyroptosis is mediated by inflammatory caspase-1/4/5/11, whereas apoptosis is mediated by apoptotic caspase-2/3/6/7/8/9/10. During the process of pyroptosis, the cell nuclear condensation, pore formation on the cell membrane mediated by GSDM family, cell welling and then flatting and pyroptotic body can be observed, ultimately cell lysis and causing inflammation. However, during the process of apoptosis, chromatin condensation, nuclear fragmentation, cytoplasm shrinkage, formation of apoptotic bodies and cell membrane is intact without triggering inflammation. Nevertheless, it is worth noting that there is no strict boundary between the pathways involved in inflammatory and apoptotic caspases (Galluzzi et al., 2018). For example, in GSDMD-deficient macrophages, caspase-1 activation induces apoptosis, which is associated with the Bid/caspase-9/caspase-3 pathway (Tsuchiya et al., 2019). In addition, caspase-3 and -8 can cause pyroptosis by cleaving the gasdermin family proteins in tumor cells (Wang et al., 2017; Sarhan et al., 2018). Previous studies have demonstrated an interplay between pyroptosis and apoptosis in sepsis. For example, Sarkar *et al.* found that caspase-1 could regulate splenic B cell apoptosis independently of IL-1β and IL-18 (Sarkar et al., 2006). In addition, Li *et al.* found that stimulator of interferon genes (STING) silencing displayed obvious anti-apoptotic effects in LPS-treated neonatal rat cardiomyocytes (NRCMs). However, the upregulation of NLRP3 offset the anti-apoptotic effect of STING silencing, indicating that NLRP3 activation could trigger apoptosis in LPS-treated NRCMs (Li et al., 2019). Likewise, in sepsis-associated encephalopathy, Chen *et al.* showed that Maf1 protects against LPS-induced blood–brain barrier (BBB) disruption both *in vitro* and *in vivo*. At the molecular level, the Maf1 protein suppressed the NF-κB/NLRP3 inflammatory pathway by directly binding to the promoter region of *NLRP3*, thus reducing apoptosis and inflammation. The overexpression of NLRP3 reversed the protective effects of Maf1 against apoptosis and pyroptosis. These results demonstrated that NLRP3 is involved in promoting apoptosis in sepsis (Chen et al., 2020). Moreover, in SAKI, the inhibition of NLRP3 by siNLRP3 decreased the number of apoptotic cells, as detected using flow cytometry in LPS-treated renal tubular epithelial cells (Liu et al., 2020). In summary, these studies demonstrated that in sepsis, pyroptosis could promote apoptosis, thus aggravating inflammation and organ dysfunction. However, the detailed mechanisms, which may be associated with the caspases involved and inflammation factors produced, still need to be explored. In turn, pannexin-1 has been found to promote NLRP3 activation during apoptosis, whereas cytosolic cytochrome c released during apoptosis negatively regulates NLRP3 (Shi and Kehrl, 2016; Chen et al., 2020). Although the exact effects of apoptosis on pyroptosis in sepsis remain unclear, pyroptosis and apoptosis are closely associated with each other in that they share common regulatory factors, such as ROS and caspases.

### 5.3 Interaction of pyroptosis and NETosis in sepsis

Neutrophil extracellular traps (NETs) are extracellular structures composed of chromatin fibers mixed with granule-derived antimicrobial peptides and enzymes that can trap, immobilize, and kill pathogens and activate other immune cells (Burgener and Schroder, 2020). NETs are generated and released during a distinct process of cell death called NETosis, which differs from apoptosis and necrosis and plays an essential role in the innate immune response (Gupta and Kaplan, 2016; Thiam et al., 2020). Chen *et al.* revealed that NETs could induce macrophage (Mϕ) pyroptosis in sepsis, thereby amplifying inflammation. At the molecular level, RAGE and dynamin-dependent signaling initiates NET-released HMGB1 endocytosis. HMGB1, a DAMP molecule, triggers a cascade of molecular events, including cathepsin B release from ruptured lysosomes followed by caspase-1 activation and pyroptosis (Chen et al., 2018). Furthermore, Tian *et al.* discovered that citrullinated histone H3 (CitH3) could mediate sepsis-induced lung injury by activating the caspase-1-dependent pyroptosis pathway (Tian et al., 2021). As CitH3 is a key component released from cells during NETosis, it was speculated that NETosis could regulate pyroptosis. Recently, Silva *et al*. found that the inhibition of GSDMD with disulfiram abrogated NET formation and reduced multiple organ dysfunction and sepsis lethality. Mechanistically, the activation of the caspase-11/GSDMD pathway controls NET release by neutrophils during sepsis (Silva et al., 2021). In summary, pyroptosis and NETosis can be reciprocally promoted in sepsis and may play a crucial role in its regulation.

Necroptosis and ferroptosis may also interact with pyroptosis during sepsis. For instance, Chen *et al.* found that receptor-interacting serine/threonine-protein kinase 3-mediated necroptosis and GSDMD-mediated pyroptosis could collaborate to amplify inflammatory signaling and enhance tissue injury during sepsis (Chen et al., 2020a). In addition, glutathione peroxidase-4 was found to play a protective role by modulating both ferroptosis and pyroptosis in bacterial infections and polymicrobial sepsis, suggesting that they may be potential therapeutic targets for treating sepsis (Zhu et al., 2019).

In conclusion, pyroptosis and other PCD pathways interact with each other and do not occur independently. The interactions between PCD pathways regulate immune and inflammation reactions, sepsis, and SAOD. Thus, identifying the key molecules and pathways involved in their interactions may provide valuable targets for treatments.

## 6. Clinical significance and further prospects of pyroptosis in sepsis

Pyroptosis, a new type of PCD pathway, has been gradually recognized in recent years, and its role in various diseases has attracted increasing attention. However, the complicated molecular mechanisms of pyroptosis and its functions in the occurrence and development of different diseases remain unclear and need to be further explored. At present, existing studies exploring the influence of pyroptosis in diseases are mainly performed at the cellular and animal experimental levels. Few clinical studies have been performed, and the specific mechanisms of pyroptosis remain elusive. Therefore, further studies are warranted to reveal the mechanisms of pyroptosis and its relationship with diseases to provide effective strategies for clinical prevention and treatment. The studies discussed in this review strongly demonstrate that pyroptosis and its related pathways play critical roles in sepsis and SAOD, although many questions remain unsolved. Previous studies have shown that inhibiting pyroptosis exerts protective effects on SAOD and may be a promising approach for the clinical treatment of SAOD. However, studies focusing on targeting pyroptosis as a therapy are still limited to the experimental level. Further clinical studies should be conducted to determine the effects of pyroptosis and possibility of its clinical application. Furthermore, studies on the involvement of ncRNAs and exosomes in pyroptosis in the regulation of sepsis may provide insights for future research, as they may be potential diagnostic or therapeutic targets. Pyroptosis may provide a novel approach for the diagnosis and treatment of sepsis, although there are still many challenges to be overcome.

### 6.1 Pyroptosis-related proteins as indicators to evaluate the severity of sepsis and SAODs

According to the Surviving Sepsis Campaign: International Guidelines for the Management of Sepsis and Septic Shock 2021, sepsis is one of the leading causes of death in both pediatric and adult intensive care units worldwide. Moreover, the prognosis of patients with sepsis accompanied by organ dysfunction are even worse. Early diagnosis is essential for implementing treatments that prevent the progression of sepsis and reduce mortality (Evans et al., 2021). As current studies reported, the changes of pyroptosis-related proteins are detected in different organs based on septic animal models and/or in blood samples from patients with sepsis, indicating that these proteins are related with sepsis and the dysfunction of organs. Thus, it is speculated that pyroptosis-related proteins may have potential roles to help diagnose sepsis or predict the severity of sepsis and its related organ dysfunction, which may be used with other serum biomarkers at the same time. Previous studies have shown that pyroptosis-related indicators can serve as markers for diagnosing and predicting the development of sepsis. For instance, Watany *et al.* measured the serum levels of IL-31, IL-1β, and NLRP3 in 149 participants (38 with sepsis; 51 with systemic inflammatory response syndrome; 30 with septic shock; and 30 healthy controls) in a case-control study. The results demonstrated that the three biomarkers could be independent prognostic biomarkers. A panel of combined markers further yielded 100% sensitivity and specificity, suggesting that the combination of IL-31, IL-1β, and NLRP3 may provide a promising diagnostic and prognostic panel for sepsis (Watany et al., 2020). In addition, Cui *et al.* reported that the absolute number and percentage of ASC-speck^+^ monocytes increased on days 6 and 7 of sepsis, and patients with lower absolute numbers of ASC-speck^+^ monocytes on day 6 showed poor 90-day survival (Cui et al., 2020). In addition, a prospective study involving 60 trauma patients and 10 healthy controls revealed that the pyroptosis of PBMCs was a potential marker for predicting the development of sepsis after trauma (Wang et al., 2018). Furthermore, Lu *et al.* discovered that the 29940 G-to-C mutation within the NLRP3 3′-UTR was a gain-of-function alteration which ultimately protected patients against susceptibility to sepsis progression and poor prognosis. At the molecular level, the G-to-C mutant could bind with miR-146a, thus suppressing NLRP3 expression and downstream inflammatory cytokine production (Lu et al., 2021). These studies demonstrate that pyroptosis-related indicators may be potential biomarkers for the diagnosis and prediction of sepsis. In the future, studies should be conducted to dynamically monitor the expression level of these proteins in animal and clinical samples, in order to find the best time point to detect the proteins. Furthermore, correlation analysis should be made between the pyroptosis-related indicators and other biomarkers, so as to explore whether these indicators are useful in the diagnosis of sepsis and sepsis-associated organ dysfunction.

### 6.2 Pyroptosis as a therapeutic target in sepsis

Although the mortality rate of sepsis has decreased with the advancement of medical technologies, sepsis and septic shock remain major healthcare problems. Epidemiological studies have reported that sepsis and septic shock affect millions of people worldwide every year, resulting in one-third to one-sixth of all deaths (Fleischmann et al., 2016; Rhee et al., 2017; Fleischmann-Struzek et al., 2020). Furthermore, the prognosis of patients with SAOD is worse. Thus, effective therapies are critically important in the treatment of sepsis. At present, the clinical treatments of sepsis are mainly comprehensive, such as fluid resuscitation and antibiotic administration. However, given the heterogeneity of individuals, individualized and precise treatments are needed to further improve the cure rate of sepsis and prognoses of patients. Pyroptosis, which is involved in inflammation and immune regulation, provides novel insights for treating inflammation and immune-related diseases, including sepsis (Chen et al., 2021; Wang et al., 2020). For example, Chen *et al.* discovered that nitrosonisoldipine (NTS) protected against sepsis in mice by inhibiting both the canonical and non-canonical inflammasome pathways. Mechanistically, NTS directly inhibited the enzymatic activities of inflammatory caspase-1, -4, and -11. Furthermore, NTS was safe for intraperitoneal administration, suggesting that targeting pyroptosis may be useful for treating sepsis (Chen et al., 2021). Notably, there are many intervention targets for regulating pyroptosis, including the activation and assembly of inflammasomes and cleaving of pro-caspases and gasdermins. For instance, Fu *et al.* showed that the NLRP3 inhibitor MCC950 and caspase-1 inhibitor Ac-YVAD-CMK ameliorated increased hippocampal neuronal pyroptosis and reduced the levels of pro-inflammatory cytokines and rescued cognitive deficits in mice with sepsis-associated encephalopathy (Fu et al., 2019). Besides, there are several drugs reported to target GSDMD directly and block pyroptosis, including necrosulfonamide (NSA), disulfiram and DL-3-n-butylphthalide (Rathkey et al., 2018; Hu et al., 2020; Han et al., 2021). For instance, Rathkey *et al.* reported that NSA treatment attenuated release of inflammatory cytokine and significantly prolonged survival in a mouse model of sepsis, providing the basis for developing novel therapies for sepsis and other inflammatory diseases (Rathkey et al., 2018). Interestingly, the involvement of ncRNAs and exosomes in regulating pyroptosis in sepsis has provided novel insights for developing precise treatments. For example, Jiao *et al.* found that neutrophil-derived exosomal miR-30d-5p could lead to macrophage pyroptosis by activating NF-κB, ultimately aggravating sepsis-induced acute lung injury (Jiao et al., 2021). In addition, Juan *et al.* revealed that M1 macrophage-derived exosomal miR-93-5p could improve SAKI by inhibiting pyroptosis in renal epithelial cells by directly targeting TXNIP (Juan et al., 2021). Exosomes are a type of natural carriers that are promising therapeutic tools. NcRNAs can interact with proteins and participate in regulating various pathways, which represent a large number of potential therapeutic targets. These characteristics provide a basis for developing precise treatments. Unfortunately, at present, studies are limited to the cellular and pre-clinical levels. Furthermore, appropriate pyroptosis-associated targets and the most relevant ncRNAs and exosomes are not well understood and remain to be explored, especially in animal experiments *in vivo* and clinical trials.

### 6.3 Further prospects of pyroptosis in sepsis

Pyroptosis plays important roles in the pathogenesis, progression, and prognosis of sepsis and is a potential diagnostic and predictive marker and promising therapeutic target. However, many questions remain to be answered before its clinical application. Among them, the most important and urgent problem is the lack of a thorough and comprehensive understanding of pyroptosis in sepsis and SAOD. Considering that pyroptosis can protect or aggravate sepsis, clarifying the role of pyroptosis in a specific state may facilitate providing positive or negative interventions. In the future, it will be necessary to carry out a large number of studies to continuously monitor the changes in the expression of pyroptosis-related indicators. The trends of clinical diagnoses and prognoses should also be comprehensively analyzed to investigate the criteria for evaluating the effect of pyroptosis and guide treatments. Furthermore, the molecular mechanisms of pyroptosis are complicated. For example, in addition to the NLRP3 inflammasome, NLR family CARD domain-containing protein 4 and absent in melanoma 2 inflammasomes can also mediate the canonical pyroptosis pathway, while the members of the gasdermin family, such as GSDME, can be cleaved and activated, subsequently forming pores on cell membranes (Guo et al., 2018). Exploring the pyroptosis-associated molecules that play leading roles in different cells under various stimuli will therefore provide the basis for treating sepsis and SAOD by regulating pyroptosis. Gene editing technologies, such as knockout or knockin of proteins *in vivo*, may be helpful in achieving this goal. Since pyroptosis and other PCD pathways control the fate of cells together, exploring their interactions and finding potential key molecules are of great significance to ensure a better outcome in pyroptosis after intervention. In addition, studies have shown that GSDMD can form pores in the mitochondrial membrane, suggesting that pyroptosis is involved in energy and metabolic regulation. Gene knockdown and/or overexpression, combined with metabolomics and proteomics, may provide novel insights into these potential functions. Finally, most interventions for pyroptosis in existing studies have poor specificity. In addition to a few studies using specific inhibitors, such as MCC950 and Ac-YVAD-CMK, most studies have shown regulation of pyroptosis by influencing the NF-κB pathway and production of ROS. In addition, investigating the modulation of transcription factors, PTMs, ncRNAs, and epigenetics on key molecules in the pyroptosis pathways using transcriptomics, methylation modifications, and PTM omics may provide novel clues for developing targeted drugs. There is no doubt that pyroptosis is a promising therapeutic strategy for sepsis; however, further studies are warranted to confirm this possibility.

## 7. Conclusion and future prospects

Since the discovery of pyroptosis, its understanding has become increasingly comprehensive and profound. In recent years, many studies have shown that pyroptosis participates in various diseases, including sepsis. This review contributes to a better understanding of the role and mechanisms of pyroptosis in sepsis and SAOD. The studies described herein provide strong evidence that pyroptosis plays an important role in sepsis; however, the detailed molecular mechanisms of pyroptosis are not well understood. Future research should focus on whether the canonical or non-canonical pyroptosis pathways are dominant in various cells under different stimuli and the inflammasomes that are involved in the canonical pyroptosis pathway. Recent advancements in technologies for gene knockout and knockin may facilitate the elucidation of the specific molecular mechanisms of pyroptosis in sepsis, providing new perspectives for the pathogenesis of sepsis and targets for treatments.

Considering that the role of pyroptosis varies in different stages of sepsis, it will be of great significance to pay attention to the mechanisms that regulate pyroptosis at multiple levels, such as epigenetics, transcription, and post-transcription. These mechanisms will provide further evidence for pyroptosis as a new factor in sepsis treatments. The application of omics technologies, such as proteomics, PTM omics, and bioinformatics, may offer clues for exploring the effects of these regulatory factors on pyroptosis and guide future experiments. In addition, further studies are needed before pyroptosis-related indicators can be used as biomarkers. The interactions between PCD pathways affect the fate of cells as well as inflammatory and immune responses. Exploring these potential links and their effects on sepsis will help develop novel treatment targets. Furthermore, exosomes carrying inflammasome components or ncRNAs can mediate the interactions between peripheral immune cells and organ-resident immune cells, as well as immune cells and organs, which may provide further potential therapeutic strategies.

Determining the role of pyroptosis in sepsis will provide new insights into its pathogenesis, diagnosis, and treatment. However, much research, especially clinical research, is needed to explore and identify the accurate molecular mechanisms and clinical value of pyroptosis in sepsis. This will contribute to revealing the diagnostic and therapeutic value of pyroptosis in sepsis. Future studies on the involvement of pyroptosis in sepsis may assess the following:

1) Detecting changes in severity and exploring the role of pyroptosis in all stages of sepsis and SAOD;
2) Involvement of various pyroptosis pathways in sepsis and SAOD;
3) Factors regulating pyroptosis at multiple levels;
4) Interactions among pyroptosis and other PCD pathways in sepsis;
5) Verifying pyroptosis-associated biomarkers for the early diagnosis of sepsis;
6) Experiments *in vivo* and clinical trials to explore therapeutic targets based on pyroptosis.

## Author contributions

All authors participated in drafting or revising of the manuscript and approved the final manuscript.

## Funding

This work was supported by the National Natural Science Foundation of China (82102254 to T-NZ and 82002021 to NY), the Doctoral Start-up Foundation of Liaoning Province (2021-BS-107 to T-NZ), and 345 Talent Program of Shengjing Hospital of China Medical University (M0691 to T-NZ and M0959 to NY).

## Acknowledgments

All figures are created with BioRender.com.

## Conflict of Interest

The authors declare that the research was conducted in the absence of any commercial or financial relationships that could be construed as a potential conflict of interest.

## Publisher’s note

All claims expressed in this article are solely those of the authors and do not necessarily represent those of their affiliated organizations, or those of the publisher, the editors and the reviewers. Any product that may be evaluated in this article, or claim that may be made by its manufacturer, is not guaranteed or endorsed by the publisher.

## Supplementary material

Supplementary Figure 1 Expression of the gasdermins family in different organs.
