# Supplementary material for: Molecular mechanisms and functions of pyroptosis in sepsis and sepsis-associated organ dysfunction
Source: Front Cell Infect Microbiol. 2022 Jul 29;12:962139. doi: 10.3389/fcimb.2022.962139 (PMC9372372; doi:10.3389/fcimb.2022.962139)
Supplement: Supplementary file 1 [file Table_1.docx]

**Supplement Table 1.** **The Role of Pyroptosis in Various Organs in Sepsis**

| **Organs** | **Name** | **Year** | **Samples** | | | **Pyroptosis-associated molecules** | **Role of pyroptosis(Injury/Protection)** | **Key points of the studies** | **REF** |
| --- | --- | --- | --- | --- | --- | --- | --- | --- | --- |
|  |  |  | **Clinical samples** | **Animal** | **Cell** |  |  |  |  |
| Heart | Wei *et al.* | 2022 | - | LPS-induced mice | - | NLRP3 , caspase-1 p20, IL-1β | Injury | Disulfiram inhibits oxidative stress and NLRP3 inflammasome activation to  prevent LPS-induced cardiac injury | (Wei et al., 2022) |
| Heart | Teng *et al.* | 2022 | - | CLP induced- mice | LPS-induced mouse HL-1 cardiomyocytes (HL-1 cells) | NLRP3, caspase1, GSDMD, IL-1β, IL-18 | Injury | Nrf2 promoted CTRP1 expression via binding to the CTRP1 promotor to inhibit  cardiomyocyte pyroptosis | (Teng et al., 2022) |
| Heart | Busch *et al.* | 2021 | - | CLP-induced WT mice and NLRP3 knockout(KO) mice | IL-1β-induced ventricular cardiomyocytes isolated from the hearts of  adult Wistar rats, IL-1β or TNF-α induced H9c2 cells | NLRP3, IL-1β | Injury | Inhibition of the NLRP3/IL-1β axis protects against  sepsis-induced cardiomyopathy | (Busch et al., 2021) |
| Heart | Qiu *et al.* | 2021 | - | CLP-induced rats | - | NLRP3, caspase‑1, IL‑1β | Injury | Ulinastatin protects against sepsis-induced myocardial  injury by inhibiting NLRP3 inflammasome activation | (Qiu et al., 2021) |
| Heart | Shao *et al.* | 2021 | - | LPS-induced mice | LPS-induced H9c2 cells | NLRP3, caspase-1, IL-1β | Injury | Gastrodin alleviates inflammatory injury of cardiomyocytes in septic  shock mice via inhibiting NLRP3 expression | (Shao et al., 2021) |
| Heart | Li *et al.* | 2021 | - | LPS-induced rats | LPS-induced H9c2 cells | NLRP3, caspase-1, IL-1β | Injury | NLRP3 inhibitor MCC950 protects  sepsis-induced myocardial dysfunction | (Li et al., 2021) |
| Heart | Wang *et al.* | 2021 | - | LPS-induced mice | H_2_O_2_-induced primary monocyte cell lines isolated from the blood  samples collected from three sham and SIMD mice, CD63+  exosome-cocultured mouse macrophage cell line RAW264.7 | NLRP3, caspase-1, IL-1β, IL-18 | Injury | Inhibiting TXNIP-NLRP3 interaction  Suppresses inflammation in sepsis-induced myocardial  dysfunction | (Wang et al., 2021) |
| Heart | Dai *et al.* | 2021 | - | LPS-induced wild-type (WT) mice and GSDMD^-^/^-^ mice | LPS induced H9ce cells and primary cardiomyocytes | NLRP3 ,cleaved caspase-1,GSDMD and  IL-1β | Injury | GSDMD mediates LPS-induced septic  myocardial dysfunction by regulating  NLRP3  inflammasome activation | (Dai, Ye, and Zhong et al., 2021) |
| Heart | Li *et al.* | 2021 | - | LPS-induced mice | LPS-induced H9c2 cells | NLRP3, caspase-1, GSDMD and IL-1β | Injury | Irisin mitigates myocardial dysfunction in sepsis by blocking the TLR4 and NLRP3 inflammasome signalings | (Li and Zhang et al., 2021) |
| Heart | Feng *et al.* | 2021 | - | - | LPS-induced H9c2 cells | NLRP3, pro-caspase-1 ,caspase‑1 p20 and IL-1β | Injury | DDX3X deficiency alleviates LPS‑induced H9c2 cardiomyocytes  pyroptosis | (Feng et al., 2021) |
| Heart | Dai *et al.* | 2021 | - | LPS-induced mice | LPS-induced primary cardiomyocytes and H9c2 cells | NLRP3,pro-caspase-1,caspase-1 p10 , GSDMD , IL-1β and IL-18 | Injury | Emodin alleviates LPS-induced myocardial injury through  inhibition of NLRP3 inflammasome activation | (Dai, Ye, and Chen et al., 2021) |
| Heart | Su *et al.* | 2021 | - | - | LPS-induced H9c2 cells | NLRP3, caspase-1,  and GSDMD | Injury | Melatonin alleviates LPS-induced myocardial injury  by inhibiting inflammation and pyroptosis | (Su et al., 2021) |
| Heart | Wei *et al.* | 2021 | - | CLP induced- mice | LPS-induced rat embryonic cardiomyoblast-derived cells, H9c2 cells | NLRP3, caspase-1, GSDMD | Injury | Syringaresinol ameliorates SICD via the ER/SIRT1/NLRP3/GSDMD pathway | (Wei et al., 2021) |
| Heart | An *et al.* | 2021 | Serums of 22 patients with sepsis-induced myocardial injury (SIMI) and 24 healthy controls | LPS-induced rat | LPS-induced H9c2 cells | Cleaved-caspase1, ASC  , NLRP3, IL-1β and IL-18 | Injury | lncRNA ZFAS1/miR-138–5p/SESN2 ameliorates sepsis-induced cardiomyocyte pyroptosis | (An et al., 2021) |
| Heart | Wang *et al.* | 2021 | - | CLP induced- rat | LPS-induced H9c2 cells | NLRP3, ASC, cleaved caspase-1, IL-1β and IL-18 | Injury | lncRNA XIST/miR-150-5p/c-Fos axis regulates sepsis-induced  myocardial injury via TXNIP-modulated pyroptosis | (Wang and Li et al., 2021) |
| Heart | Rahim *et al.* | 2021 | - | CLP induced-NLRP3^+^/^+^ and NLRP^-^/^-^ mice | - | NLRP3 | Injury | Melatonin reduced myocardial damage during sepsis by enhancing Nrf2 signaling to reduce  NLRP3 inflammasome and mitochondrial oxidative damage | (Rahim et al., 2021) |
| Heart | Liu *et al.* | 2020 | - | CLP induced- mice | LPS induced primary cardiomyocytes | NLRP3,  caspase 1, and IL-1β | Injury | SP1/LncRNA ZFAS1 /miR-590-3p/ -mediated autophagy and pyroptosis  aggravates sepsis-induced cardiac dysfunction | (Liu et al., 2020) |
| Heart | Guo *et al.* | 2020 | - | LPS-induced mice | LPS-induced H9c2 cells | NLRP3, pro-caspase-1, cleaved caspase-1, IL-1β and IL18 | Injury | Shikonin ameliorates  LPS-induced cardiac dysfunction by inhibiting NLRP3  inflammasomes | (Guo et al., 2020) |
| Heart | Li *et al.* | 2019 | - | LPS-induced mice | LPS-induced neonatal rat cardiomyocytes | NLRP3, caspase-1, IL-1β and IL-18 | Injury | STING-IRF3 contributes to LPS-induced cardiac dysfunction by activating NLRP3 | (Li N. et al., 2019) |
| Heart | Qiu *et al.* | 2019 | - | - | LPS-induced HG and H/R treated H9c2 cells | NLRP3, ASC, cleaved caspase-1 (p10), IL-1β, IL-18 | Injury | LPS aggravates HG- and H/R-induced H9C2 cell injury by inducing NLRP3  inflammasome-mediated pyroptosis | (Qiu et al., 2019) |
| Heart | Yang *et al.* | 2019 | - | LPS-induced rats | LPS-induced H9c2 cells | NLRP3, cleaved caspase-1 and caspase-1, IL-1β and IL-18 | Injury |  | (Yang et al., 2019) |
| Heart | Yang *et al.* | 2018 | - | CLP induced- rat | LPS-induced primary neonatal rat  cardiac ventricular myocytes | NLRP3, caspase-1 | Injury | SO_2_ attenuates sepsis-induced cardiac dysfunction via inhibition of NLRP3 inflammasome activation | (Yang et al., 2018) |
| Heart | Zhang *et al.* | 2017 | - | LPS-induced WT mice , NLRP3^-^/^-^mice | LPS/ATP-induced cardiac fibroblasts(CF), cardiomyocytes (CM) were challenged by supernatants of LPS/ATP-stimulated CF or a cytokine  mixture containing IL-1b, IL-18, and HMGB1 | Pro-caspase-1, caspase-1 p20, NLRP3, pro-IL-1β, IL-1β, IL-18 | Injury | CORM-3 inhibits NLRP3 inflammasome  activation in cardiac fibroblasts | (Zhang W. et al., 2017) |
| Heart | Kalbitz *et al.* | 2016 | - | CLP-induced WT mice and NLRP3^-^/^-^ mice | CMs exposed to LPS followed by ATP or nigericin, or recombinant C5a | NLRP3, caspase-1,  , ASC, IL-1β | Injury | Complement-induced activation of the cardiac NLRP3  inflammasomee contributes to the cardiomyopathy of polymicrobial sepsis | (Kalbitz et al., 2016) |
| Heart | Zhang *et al.* | 2015 | - | CLP induced- mice | LPS-induced primary neonatal cardiac fibroblasts | NLRP3, ASC, caspase-1, IL-1β | Injury | Cortistatin inhibits NLRP3 inflammasome activation of cardiac fibroblasts during  sepsis | (Zhang et al., 2015) |
| Heart | Zhang *et al.* | 2014 | - | LPS-induced mice,feces-induced  peritonitis mice | LPS-induced CFs | NLRP3, pro-caspase-1, pro-IL-1β, caspase-1 p10, IL-1β | Injury | activation of  NLRP3 inflammasome in CFs induces myocardial  dysfunction in mice with sepsis | (Zhang et al., 2014) |
| Lung | Cao *et al.* | 2022 | - | CLP-induced mice | - | NLRP3, caspase-1, GSDMD, IL-1β and IL-18 | Injury | Both pyroptosis and ferroptosis participate in CLP induced acute lung injury (ALI) | (Cao et al., 2022) |
| Lung | Liu *et al.* | 2022 | - | LPS-induced mice | LPS-induced hominine monocytic THP-1 cells | NLRP3, caspase-1 p20, ASC , GSDMD-N, IL-1β and IL-18 | Injury | Buformin alleviates sepsis-induced ALI  via inhibiting NLRP3-mediated pyroptosis | (Liu et al., 2022) |
| Lung | Li *et al.* | 2022 | - | LPS-induced WT or NLRP3^−^/^−^ micemice | Human myeloid leukemia mononuclear cells (THP-1) cell lines, rimary peritoneal macrophages  from mice | NLRP3, ASC, caspase-1, GSDMD, IL-1β and NEK7 | Injury | 1,2-diol alleviates LPS-Induced ALI through inhibiting the  NLRP3 inflammasome | (Li J. et al., 2022) |
| Lung | Zhang *et al.* | 2021 | - | CLP-induced mice | - | caspase-11, caspase-1, GSDMD and IL-1β | Injury | Luteolin alleviates lung  injury and attenuates caspase-11/1-dependent pyroptosis in the sepsis-induced lung injury | (Zhang Z. T. et al., 2021) |
| Lung | Zhou *et al.* | 2021 | - | CLP-induced mice | LPS-induced RAW 264.7 cells | NLRP3, ASC,  GSDMD and caspase-1 p20 | Injury | Syringaresinol resisted sepsis-induced ALI by suppressing pyroptosis | (Zhuo et al., 2022) |
| Lung | Li *et al.* | 2021 | - | CLP-induced mice | LPS-induced mouse alveolar macrophages MH-S | NLRP3, ASC, caspase-1, IL-1β and IL-18 | Injury | GGPPS1 knockdown suppresses NLRP3 inflammasome activity and  Attenuates sepsis-induced ALI | (Li and Li et al., 2021) |
| Lung | Zhang *et al.* | 2021 | - | CLP-induced mice | LPS-treated Raw 264.7 cells and primary murine peritoneal macrophages | NLRP3, caspase-1 p20 , IL-1β and IL-18 | Injury | Loganin alleviates sepsis-induced ALI by regulating  macrophage polarization and inhibiting NLRP3 inflammasome activation | (Zhang J. and Wang C. et al., 2021) |
| Lung | Chen *et al.* | 2021 | - | CLP-induced rats | LPS-induced Type II alveolar epithelial cells (AEC-II) | NLRP3 | Injury | Calycosin  alleviates sepsis-induced ALI by inhibiting the HMGB1/MyD88/NF-κB pathway  and NLRP3 inflammasome activation | (Chen et al.,2021) |
| Lung | Jiao *et al.* | 2021 | - | CLP-inducedmice | TNF-α-induced Polymorphonuclear neutrophils (PMNs) from healthy C57BL/6J mice, mouse Raw264.7 macrophages or bone marrow-derived macrophages (BMDMs) co-cultured with PMN-derived exosomes | NLRP3, caspase-1, GSDMD and IL-1β | Injury | miR-30d-5p from PMNs contributed to sepsisrelated ALI by inducing M1 macrophage polarization and priming macrophage pyroptosis | (Jiao et al., 2021) |
| Lung | Liu *et al.* | 2021 | - | LPS-induced mice | LPS-induced alveolar macrophages | NLRP3, ASC, caspase-1 p20, IL-  18, IL-1b, GSDMD full  Length (FL) and GSDMD-N terminal segment | Injury | WHSC1 facilitated alveolar macrophage pyroptosis in sepsis-induced ALI through NEK7-mediated NLRP3 inflammasome activation | (Liu C. et al., 2021) |
| Lung | Liu *et al.* | 2021 | - | CLP-induced mice | LPS-induced mouse monocyte/macrophage cell line  J774.A1 | Caspase-1, GSDMD, IL-1β | Injury | GPA Peptide Attenuates Sepsis-Induced ALI via Inhibiting Oxidative Stress and Pyroptosis | (Liu Y. et al., 2021) |
| Lung | Tian *et al.* | 2021 | Healthy controls, sepsis patients with ARDS and sepsis  patients without ARDS | CLP-induced mice | CitH3-induced bone marrow derived macrophages (BMDMs  ) and bone marrow derived  dendritic cells (BMDCs  ) | Caspase-1, IL-1β and IL-18 | Injury | CitH3 mediates  sepsis-induced lung injury through  activating Caspase-1 dependent  inflammasome pathway | (Tian et al., 2021) |
| Lung | Xia *et al.* | 2021 | - | CLP/LPS-induced mice | LPS-induced BMDMs | NLRP3, ASC, caspase-1, IL-1β and IL-18 | Injury | Calycosin alleviates sepsis-induced  ALI via the Inhibition of  Mitochondrial ROS-Mediated  Inflammasome Activation | (Xia et al., 2021) |
| Lung | Wang *et al.* | 2021 | - | CLP-induced mice | LPS-induced A549 cells | NLRP3, ASC, IL-1β and IL-18 | Injury | LBH alleviates sepsis-induced ALI by inhibiting  inflammation and NLRP3 inflammasome | (Wang and Shi et al., 2021) |
| Lung | Li *et al.* | 2021 | - | LPS-induced mice | LPS-induced J774A.1 cells | NLRP3, GSDMD, IL-1β and IL-18 | Injury | Mangiferin mitigates LPS-induced lung injury by inhibiting NLRP3 inflammasome activation | (Li and Xiong et al., 2021) |
| Lung | Wang *et al.* | 2021 | - | - | LPS-induced Human bronchial epithelial (Beas-2B) cells | Caspase-4, cleaved-caspase 1, GSDMD, IL-1β and IL-18 | Injury | IGF2BP2 knockdown inhibits LPS-induced pyroptosis in BEAS-2B cells by targeting caspase 4 | (Wang and Yuan et al., 2021) |
| Lung | Wang *et al.* | 2021 | - | LPS-induced mice | LPS-induced human lung microvascular endothelial cells (HLMVEC) | NLRP3, caspase-1,  cleaved caspase-1 and IL-1β | Injury | Mitochondrial redistribution of uncoupled eNOS is involved in the activation of the inflammatory response in ALI | (Wang and Sun et al., 2021) |
| Lung | Mohamed *et al.* | 2021 | - | LPS-induced mice | - | NLRP3, caspase-1 and IL-1β | Injury | Terretonin protects against Sepsis-induced ALI by regulating  SIRT1/Nrf2/N-κB p65/NLRP3 signaling | (Mohamed et al., 2021) |
| Lung | Li *et al.* | 2021 | - | CLP-induced mice | LPS-induced RAW 264.7 marophages | NLRP3, ASC, caspase-1 and IL-1β | Injury | GYY4137 alleviates sepsis-induced ALI by inhibiting  the PDGFRβ/Akt/NF-κB/NLRP3 pathway | (Li and Ma et al., 2021) |
| Lung | Zhang *et al.* | 2020 | - | LPS-induced mice | LPS and ATP -stimulated BMDMs; alveolar macrophages isolated from bronchoalveolar lavage fluid (BALF) | NLRP3,  cleaved caspase-1 and IL-1β | Injury | Cyclic helix B peptide alleviates sepsis-induced ALI by  downregulating NLRP3 inflammasome activation | (Zhang X. P. et al., 2020) |
| Lung | Li *et al*. | 2020 | Peripheral blood samples from patients with pneumoniainduced sepsis and healthy subjects | Klebsiella pneumoniae-induced rats | Peripheral blood mononuclear cells  (PBMCs) | NLRP3, NLRC4, cleaved caspase-1, cleaved GSDMD, IL-1β and IL-18 | Injury | Activation of the IL-17 signaling pathway promotes pyroptosis in  pneumonia-induced sepsis | (Li et al., 2020) |
| Lung | Zhong *et al.* | 2020 | Peripheral blood mononuclear cells were isolated from septic patients and healthy volunteers | CLP-induced wild-type, Casp1^−^/^−^,  NLRP3^−^/^−^, and SphK1^−^/^−^mice | LPS-induced BMDMs from wild-type, Casp1^−^/^−^, NLRP3^−^/^−^, and SphK1^−^/^−^mice | NLRP3, ASC, pro-caspase-1, caspase-1 p20, pro-IL-1β and IL-1β p17 | Injury | Inhibition of Sphingosine Kinase 1 Attenuates Sepsis-induced Microvascular Leakage via Inhibiting Macrophage NLRP3 Inflammasome Activation | (Zhong M. et al., 2020) |
| Lung | Zhang *et al.* | 2020 | - | CLP-induced mice | LPS-induced normal human bronchial epithelial (NHBE) cell line | NLRP3, ASC and caspase-1 | Injury | S100A12 promotes inflammation and cell apoptosis  by activating NLRP3 inflammasome signaling in sepsis-induced ARDS | (Zhang Z. et al., 2020) |
| Lung | Wang *et al.* | 2019 | - | CLP-induced mice | - | NLRP3, ASC, caspase-1, gasdermin D , IL-1β and IL-18 | Injury | Dihydromyricetin protects against CLP-induced ALI by  inhibiting NLRP3 inflammasome activation and subsequent pyroptosis | (Wang et al., 2019) |
| Lung | Lai *et al.* | 2018 | - | CLP-induced mice | Group 2 innate lymphoid cells (ILC2), LPS/TNFα-induced lung endothelial cell (EC) from WT or Caspase-1^−^/^−^ mice | Caspase-1 | Injury | Group 2 innate lymphoid cells protect lung  ECs from pyroptosis in sepsis | (Lai et al., 2018) |
| Lung | Cheng *et al.* | 2017 | - | LPS-induced Casp11^–^/^–^,  Casp1/11^DKO^, Casp1^–^/^–^ (Casp1^–^/^–^Casp11^Tg^), Casp11^fl^/^fl^ EC-specific caspase-11-knockout  mice | Human lung microvascular ECs (hMVECs), mouse lung microvascular ECs (mMVECs-L), human pulmonary artery endothelial cells (HPAECs) | Caspase 1/4/5/11, GSDMD and IL-1β | Injury | Caspase-11-mediated endothelial pyroptosis underlies endotoxemia-induced lung injury | (Cheng et al., 2017) |
| Lung | Gao *et al.* | 2015 | - | CLP-induced WT and Sirt1^-^/^-^ mice | - | Precursor and matured IL-1β | Injury | Sirt1 restrains lung inflammasome activation in a murine model of sepsis | (Gao et al., 2015) |
| Lung | Luo *et al.* | 2014 | - | CLP-induced mice | - | NLRP3, ASC, caspase-1, IL-1β and IL-18 | Injury | Hemin inhibits NLRP3 inflammasome activation in sepsis-induced ALI | (Luo et al., 2014) |
| Brain | Wang *et al.* | 2022 | - | - | LPS-induced human astrocytoma 1321N1 cells | NLRP3, pro-caspase-1, caspase-1-p20, GSDMD-N and IL-1β | Injury | Emodin relieves the inflammation and pyroptosis by regulating METTL3-mediated NLRP3 expression in LPS-treated  1321N1 cells | (Wang et al., 2022) |
| Brain | Xiao *et al.* | 2022 | - | LPS-induced WT, NLRP3 ^-^/^-^ , Asc^-^/^-^ and Gsdmd^-^/^-^ mice | LPS-induced primary microglia and astrocytes isolated from WT or  NLRP3^-^/^-^ postnatal mouse brain and primary cortical neurons obtained from E17.5 WT mice | NLRP3, ASC, GSDMD and IL-1β | Injury | NLRP3 inflammasome of microglia promotes transformation of A1 astrocytes and exacerbates neo-neuron decline, and cognitive impairment after LPS treatment | (Xiao et al., 2022) |
| Brain | Xiong *et al.* | 2022 | - | CLP-induced mice | - | NLRP3 and IL-1β | Injury | HMGB1 augments cognitive impairment in SAE by binding to MD-2 and activating  NLRP3 inflammasome | (Xiong et al., 2022) |
| Brain | Lei *et al.* | 2021 | - | CLP-induced mice | - | caspase-1, caspase-11, ASC and AIM2 | Injury | Pannexin-1 regulates neuronal  pyroptosis through autophagy in sepsis-associated encephalopathy (SAE) | (Lei et al., 2021) |
| Brain | Chen *et al.* | 2021 | - | LPS-induced mice | LPS-induced microglia | NLRP3, ASC, caspase-1,  IL-1β and IL-18 | Injury | NU9056 alleviates cognitive impairment and  emotional disorder by inhibiting the NLRP3 inflammasome in SAE model | (Chen and Qing et al., 2021) |
| Brain | Chen *et al.* | 2020 | - | CLP-induced mice | - | NLRP3, caspase 1/11, GSDMD, IL-1β and IL-18 | Injury | Sevoflurane attenuates cognitive dysfunction and  pyroptosis in septic encephalopathy model | (Chen H. et al., 2020) |
| Brain | Wang *et al.* | 2020 | - | - | LPS-induced rat pheochromocytoma PC12 cells | Cleaved caspase-1 and caspase-11, ASC, GSDMD-NT, IL-1β and IL-18 | Injury | Canonical and non-canonical pyroptosis pathways both involves in SAE cell model | (Wang Y. et al., 2020) |
| Brain | Sun *et al.* | 2020 | - | CLP-induced rats | - | NLRP3, cleaved/total caspase-1, cleaved/total GSDMD and IL-1β | Injury | P2X7 receptor mediates NLRP3/caspase-1-related pyroptosis in  SAE rat model | (Sun et al., 2020) |
| Brain | Zhong *et al.* | 2020 | - | CLP-induced WT, NLRP3^−^/^−^ and Asc^−^/^−^ mice | - | NLRP3, ASC and IL-1β | Injury | Ethyl pyruvate protects against SAE by inhibiting NLRP3 inflammasome | (Zhong X. et al., 2020) |
| Brain | Chen *et al.* | 2020 | - | LPS-induced rats | Brain microvascular endothelial cells (BMECs) , astrocytes | NLRP3, ASC, Caspas-1, GSDMD, IL-1β and IL-18 | Injury | Maf1 ameliorates SAE by inhibiting  NF-kB/NLRP3 inflammasome  signaling pathway | (Chen S. et al., 2020) |
| Brain | Xie *et al.* | 2020 | - | CLP-inducedWT and Nrf2 KO mice | - | NLRP3, ASC, caspase-1, IL-1β and IL-18 | Injury | Hydrogen attenuates SAE via inhibiting Nrf2-mediated NLRP3 pathway | (Xie K. et al., 2020) |
| Brain | Zhao *et al.* | 2020 | - | LPS-induced WT, NLRP3^−^/^−^  , and IL-1R1^−^/^−^ mice | LPS-induced primary mouse mesencephalic neuron-glial and primary mouse mixed glial | NLRP3, caspase-1and IL-1β | Injury | The role of NLRP3-generated IL-1β in  the acute-chronic transition of peripheral  LPS-induced  neuroinflammation | (Zhao et al., 2020) |
| Brain | Zhou *et al.* | 2019 | - | CLP-induced rats | - | NLRP3, caspase-1 and IL-1β | Injury | rCC16 inhibits cortical pyroptosis in sepsis | (Zhou et al., 2019) |
| Brain | Fu *et al.* | 2019 | - | CLP-induced mice | - | NLRP3, ASC, caspase-1,GSDMD, IL-1β, and IL-18 | Injury | NLRP3/Caspase-1 pyroptosis pathway mediates cognitive deficits in SAE mouse model | (Fu et al., 2019) |
| Brain | Xu *et al.* | 2019 | - | CLP-induced mice | - | Pro-caspase-1 , cleaved-caspase-1, GSDMD , GSDMD-NT, and IL-1β | Injury | Inhibiting caspase-1 protects against SAE and cognitive impairments in sepsis mouse model | (Xu et al., 2019) |
| Brain | Li *et al.* | 2019 | - | LPS-induced CYLD-KO mice | LPS and TNF-α-induced PC12 cells, astrocytes (AST) isolated from CYLD/WT or CYLD/KO mice | NLRP3, ASC, cleaved-caspase-1, cleaved GSDMD, mature IL-1β and IL-18 | Injury | CYLD deficiency exacerbates LPS  -induced  pyroptosis in astrocytes | (Li L. et al.,2019) |
| Brain | Sun *et al.* | 2019 | - | LPS-induced rats | LPS-induced human astrocyte 1321N1 cells and rat neuron PC12  cells | NLRP3, ASC, pro-caspase-1, caspase-1-p20, GSDMD and GSDMD-N | Injury | Dexmedetomidine inhibits astrocyte  pyroptosis and subsequently protects the  brain in sepsis model | (Sun et al., 2019) |
| Brain | Yu *et al.* | 2019 | - | CLP-induced mice | LPS-induced human brain microvascular  endothelial cells (HBMECs) | NLRP3, caspase-1, IL-1β and IL-18 | Injury | miRNA-200a-3p promotes sepsis‑induced  brain injury through  Keap1/Nrf2/HO1/ROS-induced NLRP3 | (Yu et al., 2019) |
| Brain | Gong *et al.* | 2019 | - | CLP-induced rats | - | NLRP3 and IL-1β | Injury | Myricitrin attenuates memory impairment in SAE  rat model  via NLRP3/Bax/Bcl  pathway | (Gong et al., 2019) |
| Brain | Sui *et al.* | 2016 | - | CLP-induced mice | LPS and ATP-induced mouse BV2 cell lines | NLRP3，pro-caspase-1 caspase-1, pro-IL-1β and IL-1β | Injury | Resveratrol improves SAE  and inhibits the NLRP3/IL-1β axis in microglia | (Sui et al., 2016) |
| Kidney | Li *et al.* | 2022 | - | CLP-induced mice | LPS-induced human kidney-2 (HK-2) cells | NLRP3, caspase-1, caspase-1 p20, GSDMD, GSDMD-NT, IL-1β and IL-18 | Injury | MIF aggravates sepsis-induced acute kidney injury (AKI) via promoting NLRP3 inflammasome mediated pyroptosis | (Li T. and Sun H. et al., 2022) |
| Kidney | Sun *et al.* | 2022 | Peripheral blood and urine collected from patients with sepsis-induced AKI and healthy volunteers | LPS-induced mice | LPS-induced HK-2 cells | NLRP3, caspase-1, GSDMD-N, IL-1β and IL-18 | Injury | USF2 knockdown reduces  pyroptosis and further ameliorates sepsis-induced AKI | (Sun et al., 2022) |
| Kidney | Li *et al.* | 2022 | - | LPS-induced rats | LPS-induced HK-2 cells | NLRP3, ASC and caspase-1, IL-1β and IL-18 | Injury | TRIM3 protects against LPS-induced AKI via inhibiting IRF3 pathway and NLRP3 infammasome activation | (Li W. and Tan Y. et al., 2022) |
| Kidney | Rodriguez *et al.* | 2021 | - | CLP-induced rats | - | NLRP3, ASC, IL-1β and IL-18 | Injury | Activated platelets may contribute to sepsis-induced renal injury | (Borges-Rodriguez et al., 2021) |
| Kidney | Li *et al.* | 2021 | - | LPS-induced mice | LPS-induced HK-2 cells | NLRP3, caspase-1, ASC, GSDMD,  GSDMD-N, IL-1β and IL-18 | Injury | miR-30c-5p alleviates pyroptosis via  targeting TXNIP  in sepsis-induced AKI | (Li and Yao et al., 2021) |
| Kidney | Wang *et al.* | 2021 | - | CLP-induced mice | LPS-induced HK-2 cells | NLRP3, ASC, cleaved-caspase-1, IL-1β and IL-18 | Injury | ROCK1 regulates sepsis-induced AKI through TLR2-mediated ERS/pyroptosis axis | (Wang and Xing et al., 2021) |
| Kidney | Ling *et al.* | 2021 | - | CLP-induced mice | LPS-induced HK-2 cells | NLRP3, caspase-1, cleaved caspase-1, ASC, GSDMD-NT, IL-1β and IL-18 | Injury | LncRNA GAS5 /miR-579-3p/SIRT1/PGC-1a/Nrf2 signaling  pathway reduces cell pyroptosis in sepsis-associated AKI | (Ling et al., 2021) |
| Kidney | Juan *et al.* | 2021 | - | CLP-induced mice | Primary peritoneal macrophages harvested from the peritoneal exudates of mice following the established protocol, LPS-induced TCMK-1 cells, a mouse kidney epithelial cell line | NLRP3, cleaved caspase-1, GSDMD, IL-1β and IL-18 | Injury | Macrophages-derived exosomal miR-93-5p regulates  TXNIP directly to influence pyroptosis in renal epithelial cells | (Juan et al., 2021) |
| Kidney | Yang *et al.* | 2021 | - | CLP-induced mice | - | NLRP1, caspase-1, IL-1β and IL-18 | Injury | Caspase-1-Inhibitor AC-YVAD-CMK ameliorates sepsis-induced AKI by inhibiting pyroptosis | (Yang et al., 2021) |
| Kidney | Deng *et al*. | 2021 | - | LPS-induced mice | LPS-induced Human primary renal TECs | Caspase-1, cleaved caspase-1, GSDMD, GSDMD-N, IL-1β and IL-18 | Injury | LncRNA MEG3/miR-18a-3p/GSDMD pathway  promotes renal tubular epithelial  cell pyroptosis in LPS-induced AKI | (Deng et al., 2021) |
| Kidney | Zhou *et al.* | 2020 | - | LPS-induced mice | - | NLRP3, ASC, cleaved-caspase-1, caspase-1, IL-1β and IL-18 | Injury | C16 ameliorated LPS-induced renal inflammation and injury | (Zhou et al., 2020) |
| Kidney | Huang *et al.* | 2020 | - | LPS-induced mice | LPS-induced HK-2 cells | NLRP3, caspase-1 and IL-1β | Injury | Inhibiting pannexin-1 prevents against sepsis-induced AKI via decreasing NLRP3 activation | (Huang G. et al., 2020) |
| Kidney | Tanuseputero *et al.* | 2020 | - | CLP-induced mice | - | NLRP3, ASC, caspase-1and IL-1β | Injury | Arginine  supplementation attenuates septic AKI, partly via inhibiting NLRP3 inflammasome | (Tanuseputero et al., 2020) |
| Kidney | Liu *et al.* | 2020 | - | LPS-induced mice | LPS-induced mouse renal tubular epithelial (TCMK-1) cells | NLRP3, cleaved-caspase-1, GSDMD, IL-1β, and IL-18 | Injury | Inhibiting DRP1 alleviates LPS-Induced AKI by inhibiting NLRP3 inflammasome activation | (Liu and Wang et al., 2020) |
| Kidney | Yang *et al.* | 2020 | - | LPS-induced rats | - | NLRP3, ASC, caspase-1, cleaved-caspase-1, IL-1β and IL-18 | Injury | Dexmedetomidine alleviates  LPS-induced AKI by inhibiting  NLRP3 Inflammasome activation | (Yang et al., 2020) |
| Kidney | Tan *et al.* | 2020 | Serum samples from sepsis patients with AKI and  healthy volunteers | - | LPS-induced HK-2 cells | NLRP3, IL-1β and IL-18 | Injury | lncRNA DLX6-AS1/ miR-223-3p/NLRP3promotes LPS-mediated  cytotoxicity and pyroptosis in HK-2 | (Tan et al., 2020) |
| Kidney | Deng *et al.* | 2020 | - | abdominal resection  through surgical suture (CELP)-induced rats | hypoxia-reoxygenation -induced renal tubular epithelial (RTE) cells obtained from rats | NLRP3, ASC, ro-caspase-1, caspase-1 p10, IL-1β and IL-18 | Injury | Activation of NLRP3 inflammasome promotes sepsis-induced AKI | (Deng et al., 2020) |
| Kidney | Yao *et al.* | 2019 | - | LPS-induced rats | - | NLRP3, ASC, pro-caspase-1, caspase-1 P20, pro-IL-1β, IL-1β and IL-18 | Injury | Dexmedetomidine attenuates LPS-induced AKI by inhibiting oxidative stress damage and NLRP3  inflammasome activation | (Yao et al., 2019) |
| Kidney | Gao *et al.* | 2019 | - | - | LPS-induced HK-2 cells | NLRP3, caspase-1, cleaved-caspase-1, IL-1β and IL-18 | Injury | SIRT1  Alleviates LPS-induced  AKI via inhibiting  NLRP3  Inflammasome activation | (Gao et al., 2019) |
| Kidney | Wang *et al.* | 2015 | - | CLP-induced rats | - | NLRP3, ASC, caspase-1 p10 and IL-1β | Injury | CORM-2 protects against sepsis-induced AKI and inhibits NLRP3 inflammasome activation | (Wang et al., 2015) |
| Liver | Li *et al.* | 2022 | - | CLP-induced mice | LPS-induced HepG2 and Hep3B cells | NLRP3, ASC, cleaved caspase-1, and mature IL-1β | Injury | PPARγ alleviates sepsis- induced liver injury by inhibiting  hepatocyte pyroptosis | (Li Z. et al., 2022) |
| Liver | Yang *et al.* | 2022 | - | LPS/(d-galactosamine)  D-GalN-induced mice | LPS-induced RAW264.7, NCTC1469 and BMDMs | NLRP3, GSDMD, GSDMD-NT and IL-1β | Injury | Maresin 1 protects against acute liver injury by inhibiting macrophage pyroptosis and  inflammatory response | (Yang et al., 2022) |
| Liver | Guo *et al.* | 2021 | - | LPS-induced mice | LPS-induced Hepatocytes and Kupffer cells isolated from mice | NLRP3, pro-caspase-1, cleaved caspase-1,  pro-IL-1β, cleaved IL-1β and IL-18 | Injury | GBP-5 aggravates  sepsis-associated liver injury via NLRP3 inflammasome  activation | (Guo et al., 2021) |
| Liver | Zhang *et al.* | 2021 | - | Escherichia coli-induced WT, CD38^-^/^-^ and CD38^-^/^-^TLR4^mut^ mice | - | NLRP3, ASC,  pro-caspase-1, cleaved caspase-1, IL-1β and IL-18 | Injury | CD38 knockout  exacerbates bacteria-induced liver damage through pyroptosis | (Zhang H. and Du Y et al., 2021) |
| Liver | Wang *et al.* | 2021 | Human serum samples  obtained from control healthy subjects and sepsis patients | LPS/CLP-induced WT and NLRP3^-^/^-^ mice | LPS-induced Raw264.7; EV-treated primary hepatocytes and AML-12 (hepatocyte cell line) | NLRP3, ASC, pro-caspase-1, cleaved caspase-1, GSDMD, pro-IL-1β and IL-1β | Injury | LPS induces HMGB1-loaded EVs released from macrophages to trigger NLRP3 inflammasome-mediated pyroptosis in  hepatocytes | (Wang and Jin et al., 2021) |
| Liver | Zhang *et al.* | 2021 | - | Alcohol-induced mice | Alcohol or LPS-induced Human normal hepatocytes (LO2 cells) | NLRP3, cleaved caspase-1, cleaved GSDMD, IL-1β and IL-18 | Injury | Butyrate ameliorates alcohol-induced liver injury and inflammation through reducing endotoxemia and  inhibiting pyroptosis | (Zhang T. et al., 2021) |
| Liver | Li *et al.* | 2021 | - | LPS-induced mice | LPS-induced RAW264.7 cells | NLRP3, caspase-1 , GSDMD and IL-1β | Injury | Irisin alleviates LPS-mediated  liver injury via inhibiting apoptosis, NLRP3 inflammasome activation and NF-jB signaling | (Li and Tan et al., 2021) |
| Liver | Pai *et al.* | 2020 | - | CLP-induced mice | - | NLRP3, caspase-1, caspase-11, GSDMD, IL-1β and IL-18 | Protection first, then injury | Glutamine modulates the balance of liver pyroptosis at in sepsis mice | (Pai et al., 2020) |
| Liver | Xu *et al.* | 2020 | - | LPS-induced mice | - | NLRP3, pro-caspase-1, cleaved caspase-1,GSDMD and GSDMD-NT | Injury | Estrogen protects against liver damage in  sepsis by inhibiting oxidative stress-mediated pyroptosis | (Xu et al., 2020) |
| Liver | Huang *et al.* | 2020 | Serum of sepsis group and normal group | CLP-induced mice | - | Caspase-1 and IL-1β | Injury | HMGB1 promotes acute liver injury in sepsis via inducing pyroptosis of liver macrophages | (Huang Y. et al., 2020) |
| Liver | Liu *et al.* | 2020 | - | LPS-inducedWT and Hspa12a^−^/^−^ mice | LPS-induced primary hepatocytes | NLRP1, NLRP3, ASC, caspase-1, caspase11 and GSDMD | Injury | HSPA12A attenuates LPS-induced liver injury  via inhibiting caspase-11-mediated hepatocyte pyroptosis | (Liu and Du S et al., 2020) |
| Liver | Miao *et al.* | 2016 | - | LPS-induced mice | LPS-induced Kupffer cell isolated from the livers of mice, mouse Bone marrow stromal cells (BMSCs) isolated from the femurs and tibias | NLRP3, ASC, pro-Casp1, caspase-1 and pro-IL-1β | Injury | BMSCs attenuate LPS-induced mouse acute liver  injury via inhibiting NLRP3  inflammasome in Kupffer cells | (Miao et al., 2016) |
| Liver | Diao *et al.* | 2014 | - | Burn + LPS-induced Rats | - | NLRP3 | Injury | Burn plus LPS augments endoplasmic reticulum stress and NLRP3 inflammasome activation and reduces PGC-1α in liver | (Diao et al., 2014) |
| Vascular endothelial cells | Liang *et al.* | 2022 | Peripheral  blood collected from sepsis patients and healthy subjects | - | endothelial  progenitor cells (EPCs) from peripheral  blood, bone marrow mesenchymal stem cells (MSCs) | NLRP3, ASC, cleaved-caspase-1 and IL-18 | Injury | lncRNA IGF2-AS regulated nucleotide metabolism by mediating HMGA1 to promote pyroptosis of EPCs in sepsis patients | (Liang et al., 2022) |
| Vascular endothelial cells | Zhao *et al.* | 2021 | - | - | LPS-induced human umbilical vein endothelial cells  (HUVECs) | NLRP3, cleaved caspase-1,  cleaved GSDMD and IL-1β | Injury | LPS induces vascular endothelial cell pyroptosis by  SP1/RCN2/ROS signaling pathway | (Zhao et al., 2021) |
| Vascular endothelial cells | Lv *et al.* | 2021 | - | CLP-induced mice | LPS-induced HUVECs | NLRP3, ASC, caspase-1 and IL-1β | Injury | SIRT3 improves CLP-induced  endothelial dysfunction by inhibiting  NF-κB and NLRP3 signaling pathways | (Lv et al., 2021) |
| Vascular endothelial cells | Hu *et al.* | 2021 | - | CLP-induced mice | IL-1β induced Human aortic endothelial cells (HAECs) | NLRP3, caspase-1 and IL-1β | Injury | NLRP3/IL-1β axis impairs vasodilation and can be inhibited by melatonin | (Hu et al., 2021) |
| Vascular endothelial cells | Luo *et al.* | 2020 | - | CLP-induced WT and NLRP3^−^/^−^ mice | LPS-induced HUVECs | NLRP3 and  IL-1β | Injury | NLRP3 inflammasome promotes endothelial dysfunction  of early sepsis in mice | (Luo et al., 2020) |
| Vascular endothelial cells | Chen *et al.* | 2019 | - | CLP-induced WT mice or transgenic (DEFA1/  DEFA3) mice | - | Pro-caspase-1and caspase-1 p10 | Injury | Increased gene copy number of DEFA1/DEFA3 exacerbates  sepsis by inducing endothelial pyroptosis | (Chen et al., 2019) |
| Intestinal barrier | Chen *et al.* | 2021 | - | LPS-induced mice | - | NLRP3, ASC, caspase 1, GSDMD, GSDME, GSDMA, IL-1β and IL-18 | Injury | JQ1 blocks  inflammatory pyroptosis-related  acute colon injury induced by LPS | (Chen and Zhong et al., 2021) |
| Intestinal barrier | Xie *et al.* | 2020 | - | CLP-induced mice | LPS-induced Caco-2 cells | NLRP3, ASC, caspase 1, IL-1β and IL-18 | Injury | AS-IV sepsis-induced intestinal barrier dysfunction via inhibiting RhoA/NLRP3 inflammasome signal pathway | (Xie S. et al., 2020) |
| Intestinal barrier | Wang *et al.* | 2020 | - | CLP-induced rats | - | caspase-1, caspase-11, GSDMD, IL-1β and IL-18 | Injury | CO inhibits intestinal mucosal pyroptosis in sepsis rat | (Wang H. et al., 2020) |
| Intestinal barrier | Zhang *et al.* | 2018 | - | LPS-induced mice | Rat intestinal epithelial cell line (IEC-6, CRL-1592) | NLRP3, caspase1 and caspase-11 | Injury | Propofol inhibits P2X7R upregulation . but doesn’t  reduce LPS-induced pyroptosis and intestinal epithelial  injury | (Zhang et al., 2018) |
| Intestinal barrier | Zhang *et al.* | 2017 | - | CLP-induced rats | - | NLRP3, ASC, pro-caspase-1, caspase-1 p10 and IL-1β | Injury | Glucose-Insulin-Potassium alleviates intestinal mucosal  barrier injuries by decreasing UCP2 and NLRP3  inflammasome in Sepsis | (Zhang J. L. et al., 2017) |

**References:**

Borges-Rodriguez, M., Shields, C. A., Travis, O. K., Tramel, R. W., Baik, C. H., and Giachelli, C. A., et al. (2021). Platelet inhibition prevents NLRP3 inflammasome activation and Sepsis-Induced kidney injury. *Int. J. Mol. Sci.* 22(19):10330. doi: 10.3390/ijms221910330

Cao, Z., Qin, H., Huang, Y., Zhao, Y., Chen, Z., and Hu, J., et al. (2022). Crosstalk of pyroptosis, ferroptosis, and mitochondrial aldehyde dehydrogenase 2-related mechanisms in sepsis-induced lung injury in a mouse model. *Bioengineered*. 13(3), 4810-4820. doi: 10.1080/21655979.2022.2033381

Chen, G., Hou, Y., Li, X., Pan, R., and Zhao, D. (2021). Sepsis-induced acute lung injury in young rats is relieved by calycosin through inactivating the HMGB1/MyD88/NF-kappaB pathway and NLRP3 inflammasome. *Int. Immunopharmacol.* 96, 107623. doi: 10.1016/j.intimp.2021.107623

Chen, H., Peng, Y., Wang, L., and Wang, X. (2020). Sevoflurane attenuates cognitive dysfunction and NLRP3-dependent caspase-1/11-GSDMD pathway-mediated pyroptosis in the hippocampus via upregulation of SIRT1 in a sepsis model. *Arch Physiol Biochem*, 1-8. doi: 10.1080/13813455.2020.1773860

Chen, L., Qing, W., Yi, Z., Lin, G., Peng, Q., and Zhou, F. (2021). NU9056, a KAT 5 inhibitor, treatment alleviates brain dysfunction by inhibiting NLRP3 inflammasome activation, affecting gut microbiota, and derived metabolites in LPS-Treated mice. *Front Nutr*. 8, 701760. doi: 10.3389/fnut.2021.701760

Dai, S., Ye, B., Chen, L., Hong, G., Zhao, G., and Lu, Z. (2021). Emodin alleviates LPS-induced myocardial injury through inhibition of NLRP3 inflammasome activation. *Phytother. Res.* 35(9), 5203-5213. doi: 10.1002/ptr.7191

Dai, S., Ye, B., Zhong, L., Chen, Y., Hong, G., and Zhao, G., et al. (2021). GSDMD mediates LPS-Induced septic myocardial dysfunction by regulating ROS-dependent NLRP3 inflammasome activation. *Front Cell Dev Biol*. 9, 779432. doi: 10.3389/fcell.2021.779432

Deng, H., Chen, F., Wang, Y., Jiang, H., Dong, Z., and Yuan, B., et al. (2020). The role of activated NLRP3 inflammatory body in acute kidney injury in rats caused by sepsis and NLRP3-TXNIP signaling pathway. *Saudi J. Biol. Sci.* 27(5), 1251-1259. doi: 10.1016/j.sjbs.2020.03.018

Diao, L., Marshall, A. H., Dai, X., Bogdanovic, E., Abdullahi, A., and Amini-Nik, S., et al. (2014). Burn plus lipopolysaccharide augments endoplasmic reticulum stress and NLRP3 inflammasome activation and reduces PGC-1alpha in liver. *Shock*. 41(2), 138-144. doi: 10.1097/SHK.0000000000000075

Feng, D., Guo, L., Liu, J., Song, Y., Ma, X., and Hu, H., et al. (2021). DDX3X deficiency alleviates LPS-induced H9c2 cardiomyocytes pyroptosis by suppressing activation of NLRP3 inflammasome. *Exp. Ther. Med.* 22(6), 1389. doi: 10.3892/etm.2021.10825

Gao, Q., and Zhu, H. (2019). The overexpression of sirtuin1 (SIRT1) alleviated lipopolysaccharide (LPS)-Induced acute kidney injury (AKI) via inhibiting the activation of Nucleotide-Binding oligomerization Domain-Like receptors (NLR) family pyrin domain containing 3 (NLRP3) inflammasome. *Med Sci Monit*. 25, 2718-2726. doi: 10.12659/MSM.913146

Gao, R., Ma, Z., Hu, Y., Chen, J., Shetty, S., and Fu, J. (2015). Sirt1 restrains lung inflammasome activation in a murine model of sepsis. *Am J Physiol Lung Cell Mol Physiol*. 308(8), L847-L853. doi: 10.1152/ajplung.00274.2014

Gong, J., Luo, S., Zhao, S., Yin, S., Li, X., and Mou, T. (2019). Myricitrin attenuates memory impairment in a rat model of sepsis-associated encephalopathy via the NLRP3/Bax/Bcl pathway. *Folia Neuropathol.* 57(4), 327-334. doi: 10.5114/fn.2019.89856

Guo, T., Jiang, Z. B., Tong, Z. Y., Zhou, Y., Chai, X. P., and Xiao, X. Z. (2020). Shikonin ameliorates LPS-Induced cardiac dysfunction by SIRT1-Dependent inhibition of NLRP3 inflammasome. *Front Physiol*. 11, 570441. doi: 10.3389/fphys.2020.570441

Hu, S., Pi, Q., Luo, M., Cheng, Z., Liang, X., and Luo, S., et al. (2021). Contribution of the NLRP3/IL-1beta axis to impaired vasodilation in sepsis through facilitation of eNOS proteolysis and the protective role of melatonin. *Int. Immunopharmacol.* 93, 107388. doi: 10.1016/j.intimp.2021.107388

Huang, G., Bao, J., Shao, X., Zhou, W., Wu, B., and Ni, Z., et al. (2020). Inhibiting pannexin-1 alleviates sepsis-induced acute kidney injury via decreasing NLRP3 inflammasome activation and cell apoptosis. *Life Sci.* 254, 117791. doi: 10.1016/j.lfs.2020.117791

Huang, Y., Zang, K., Shang, F., Guo, S., Gao, L., and Zhang, X. (2020). HMGB1 mediates acute liver injury in sepsis through pyroptosis of liver macrophages. *Int J Burns Trauma*. 10(3), 60-67

Lai, D., Tang, J., Chen, L., Fan, E. K., Scott, M. J., and Li, Y., et al. (2018). Group 2 innate lymphoid cells protect lung endothelial cells from pyroptosis in sepsis. *Cell Death Dis.* 9(3), 369. doi: 10.1038/s41419-018-0412-5

Lei, Y., Zhou, R., Sun, X., Tang, F., Gao, H., and Chen, L., et al. (2021). The pannexin-1 channel regulates pyroptosis through autophagy in a mouse model of sepsis-associated encephalopathy. *Ann Transl Med*. 9(24), 1802. doi: 10.21037/atm-21-6579

Li, J., Ma, J., Li, M., Tao, J., Chen, J., and Yao, C., et al. (2021). GYY4137 alleviates sepsis-induced acute lung injury in mice by inhibiting the PDGFRbeta/Akt/NF-kappaB/NLRP3 pathway. *Life Sci.* 271, 119192. doi: 10.1016/j.lfs.2021.119192

Li, L. L., Dai, B., Sun, Y. H., and Zhang, T. T. (2020). The activation of IL-17 signaling pathway promotes pyroptosis in pneumonia-induced sepsis. *Ann Transl Med*. 8(11), 674. doi: 10.21037/atm-19-1739

Li, L., Shu, M. Q., and Chen, J. (2019). CYLD deficiency exacerbates lipopolysaccharide (LPS)-induced pyroptosis in astrocytes of mice with sepsis. *Biochem Biophys Res Commun*. 514(4), 1066-1073. doi: 10.1016/j.bbrc.2019.05.033

Li, N., Xiong, R., He, R., Liu, B., Wang, B., and Geng, Q. (2021). Mangiferin mitigates Lipopolysaccharide-Induced lung injury by inhibiting NLRP3 inflammasome activation. *J Inflamm Res*. 14, 2289-2300. doi: 10.2147/JIR.S304492

Li, Q., Tan, Y., Chen, S., Xiao, X., Zhang, M., and Wu, Q., et al. (2021). Irisin alleviates LPS-induced liver injury and inflammation through inhibition of NLRP3 inflammasome and NF-kappaB signaling. *J Recept Signal Transduct Res*. 41(3), 294-303. doi: 10.1080/10799893.2020.1808675

Li, Q., Zhang, M., Zhao, Y., and Dong, M. (2021). Irisin protects against LPS-Stressed cardiac damage through inhibiting inflammation, apoptosis, and pyroptosis. *Shock*. 56(6), 1009-1018. doi: 10.1097/SHK.0000000000001775

Li, S., Guo, Z., and Zhang, Z. Y. (2021). Protective effects of NLRP3 inhibitor MCC950 on sepsis-induced myocardial dysfunction. *J Biol Regul Homeost Agents*. 35(1), 141-150. doi: 10.23812/20-662-A

Li, W., Tan, Y., Gao, F., and Xiang, M. (2022). Overexpression of TRIM3 protects against LPS-induced acute kidney injury via repressing IRF3 pathway and NLRP3 inflammasome. *Int. Urol. Nephrol.* 54(6), 1331-1342. doi: 10.1007/s11255-021-03017-z

Liu, J., Du S, Kong, Q., Zhang, X., Jiang, S., and Cao, X., et al. (2020). HSPA12A attenuates lipopolysaccharide-induced liver injury through inhibiting caspase-11-mediated hepatocyte pyroptosis via PGC-1alpha-dependent acyloxyacyl hydrolase expression. *Cell Death Differ.* 27(9), 2651-2667. doi: 10.1038/s41418-020-0536-x

Luo, M., Meng, J., Yan, J., Shang, F., Zhang, T., and Lv, D., et al. (2020). Role of the Nucleotide-Binding Domain-Like receptor protein 3 inflammasome in the endothelial dysfunction of early sepsis. *Inflammation*. 43(4), 1561-1571. doi: 10.1007/s10753-020-01232-x

Luo, Y. P., Jiang, L., Kang, K., Fei, D. S., Meng, X. L., and Nan, C. C., et al. (2014). Hemin inhibits NLRP3 inflammasome activation in sepsis-induced acute lung injury, involving heme oxygenase-1. *Int. Immunopharmacol.* 20(1), 24-32. doi: 10.1016/j.intimp.2014.02.017

Lv, D., Luo, M., Yan, J., Yang, X., and Luo, S. (2021). Protective Effect of Sirtuin 3 on CLP-Induced Endothelial Dysfunction of Early Sepsis by Inhibiting NF-kappaB and NLRP3 Signaling Pathways. *Inflammation*. 44(5), 1782-1792. doi: 10.1007/s10753-021-01454-7

Miao, C. M., Jiang, X. W., He, K., Li, P. Z., Liu, Z. J., and Cao, D., et al. (2016). Bone marrow stromal cells attenuate LPS-induced mouse acute liver injury via the prostaglandin E 2-dependent repression of the NLRP3 inflammasome in Kupffer cells. *Immunol. Lett.* 179, 102-113. doi: 10.1016/j.imlet.2016.09.009

Mohamed, G. A., Ibrahim, S., El-Agamy, D. S., Elsaed, W. M., Sirwi, A., and Asfour, H. Z., et al. (2021). Terretonin as a New Protective Agent against Sepsis-Induced Acute Lung Injury: Impact on SIRT1/Nrf2/NF-kappaBp65/NLRP3 Signaling. *Biology (Basel)*. 10(11). doi: 10.3390/biology10111219

Qiu, J., Xiao, X., Gao, X., and Zhang, Y. (2021). Ulinastatin protects against sepsisinduced myocardial injury by inhibiting NLRP3 inflammasome activation. *Mol. Med. Rep.* 24(4). doi: 10.3892/mmr.2021.12369

Qiu, Z., He, Y., Ming, H., Lei, S., Leng, Y., and Xia, Z. Y. (2019). Lipopolysaccharide (LPS) aggravates high glucose- and Hypoxia/Reoxygenation-Induced injury through activating ROS-Dependent NLRP3 Inflammasome-Mediated pyroptosis in H9C2 cardiomyocytes. *J. Diabetes Res.* 2019, 8151836. doi: 10.1155/2019/8151836

Shao, F., Zhou, L., Zhang, Y., Chen, H., Zhang, Y., and Guan, Z. (2021). Gastrodin alleviates inflammatory injury of cardiomyocytes in septic shock mice via inhibiting NLRP3 expression. *In Vitro Cell Dev Biol Anim*. 57(5), 571-581. doi: 10.1007/s11626-021-00593-3

Su, Z. D., Wei, X. B., Fu, Y. B., Xu, J., Wang, Z. H., and Wang, Y., et al. (2021). Melatonin alleviates lipopolysaccharide-induced myocardial injury by inhibiting inflammation and pyroptosis in cardiomyocytes. *Ann Transl Med*. 9(5), 413. doi: 10.21037/atm-20-8196

Sui, D. M., Xie, Q., Yi, W. J., Gupta, S., Yu, X. Y., and Li, J. B., et al. (2016). Resveratrol protects against Sepsis-Associated encephalopathy and inhibits the NLRP3/IL-1beta axis in microglia. *Mediators Inflamm*. 2016, 1045657. doi: 10.1155/2016/1045657

Sun, X., Zhou, R., Lei, Y., Hu, J., and Li, X. (2020). The ligand-gated ion channel P2X7 receptor mediates NLRP3/caspase-1-mediated pyroptosis in cerebral cortical neurons of juvenile rats with sepsis. *Brain Res.* 1748, 147109. doi: 10.1016/j.brainres.2020.147109

Tanuseputero, S. A., Lin, M. T., Yeh, S. L., and Yeh, C. L. (2020). Intravenous arginine administration downregulates NLRP3 inflammasome activity and attenuates acute kidney injury in mice with polymicrobial sepsis. *Mediators Inflamm*. 2020, 3201635. doi: 10.1155/2020/3201635

Wang, G., Jin, S., Huang, W., Li, Y., Wang, J., and Ling, X., et al. (2021). LPS-induced macrophage HMGB1-loaded extracellular vesicles trigger hepatocyte pyroptosis by activating the NLRP3 inflammasome. *Cell Death Discov*. 7(1), 337. doi: 10.1038/s41420-021-00729-0

Wang, H., Sun, X., Lu, Q., Zemskov, E. A., Yegambaram, M., and Wu, X., et al. (2021). The mitochondrial redistribution of eNOS is involved in lipopolysaccharide induced inflammasome activation during acute lung injury. *Redox Biol*. 41, 101878. doi: 10.1016/j.redox.2021.101878

Wang, H., Zhang, S., Zhao, H., Qin, H., Zhang, J., and Dong, J., et al. (2020). Carbon monoxide inhibits the expression of proteins associated with intestinal mucosal pyroptosis in a rat model of sepsis induced by cecal ligation and puncture. *Med Sci Monit*. 26, e920668. doi: 10.12659/MSM.920668

Wang, J., Yuan, X., and Ding, N. (2021). IGF2BP2 knockdown inhibits LPS-induced pyroptosis in BEAS-2B cells by targeting caspase 4, a crucial molecule of the non-canonical pyroptosis pathway. *Exp. Ther. Med.* 21(6), 593. doi: 10.3892/etm.2021.10025

Wang, P., Huang, J., Li, Y., Chang, R., Wu, H., and Lin, J., et al. (2015). Exogenous carbon monoxide decreases Sepsis-Induced acute kidney injury and inhibits NLRP3 inflammasome activation in rats. *Int. J. Mol. Sci.* 16(9), 20595-20608. doi: 10.3390/ijms160920595

Wang, Q. L., Xing, W., Yu, C., Gao, M., and Deng, L. T. (2021). ROCK1 regulates sepsis-induced acute kidney injury via TLR2-mediated endoplasmic reticulum stress/pyroptosis axis. *Mol. Immunol.* 138, 99-109. doi: 10.1016/j.molimm.2021.07.022

Wang, Y. C., Liu, Q. X., Zheng, Q., Liu, T., Xu, X. E., and Liu, X. H., et al. (2019). Dihydromyricetin alleviates Sepsis-Induced acute lung injury through inhibiting NLRP3 Inflammasome-Dependent pyroptosis in mice model. *Inflammation*. 42(4), 1301-1310. doi: 10.1007/s10753-019-00990-7

Wang, Y., Liu, X., Wang, Q., and Yang, X. (2020). Roles of the pyroptosis signaling pathway in a sepsis-associated encephalopathy cell model. *J. Int. Med. Res.* 48(8), 1220749319. doi: 10.1177/0300060520949767

Wang, Y., Shi, Y., Zhang, X., Fu, J., and Chen, F. (2021). Overexpression of limb bud and heart alleviates Sepsis-Induced acute lung injury via inhibiting the NLRP3 inflammasome. *Biomed Res. Int.* 2021, 4084371. doi: 10.1155/2021/4084371

Wei, A., Liu, J., Li, D., Lu, Y., Yang, L., and Zhuo, Y., et al. (2021). Syringaresinol attenuates sepsis-induced cardiac dysfunction by inhibiting inflammation and pyroptosis in mice. *Eur. J. Pharmacol.* 913, 174644. doi: 10.1016/j.ejphar.2021.174644

Wei, S., Xiao, Z., Huang, J., Peng, Z., Zhang, B., and Li, W. (2022). Disulfiram inhibits oxidative stress and NLRP3 inflammasome activation to prevent LPS-induced cardiac injury. *Int. Immunopharmacol.* 105, 108545. doi: 10.1016/j.intimp.2022.108545

Xia, Y., Cao, Y., Sun, Y., Hong, X., Tang, Y., and Yu, J., et al. (2021). Calycosin alleviates Sepsis-Induced acute lung injury via the inhibition of mitochondrial ROS-Mediated inflammasome activation. *Front Pharmacol*. 12, 690549. doi: 10.3389/fphar.2021.690549

Xiao, T., Ji, H., Shangguan, X., Qu, S., Cui, Y., and Xu, J. (2022). NLRP3 inflammasome of microglia promotes A1 astrocyte transformation, neo-neuron decline and cognition impairment in endotoxemia. *Biochem Biophys Res Commun*. 602, 1-7. doi: 10.1016/j.bbrc.2022.02.092

Xie, K., Zhang, Y., Wang, Y., Meng, X., Wang, Y., and Yu, Y., et al. (2020). Hydrogen attenuates sepsis-associated encephalopathy by NRF2 mediated NLRP3 pathway inactivation. *Inflamm. Res.* 69(7), 697-710. doi: 10.1007/s00011-020-01347-9

Xie, S., Yang, T., Wang, Z., Li, M., Ding, L., and Hu, X., et al. (2020). Astragaloside IV attenuates sepsis-induced intestinal barrier dysfunction via suppressing RhoA/NLRP3 inflammasome signaling. *Int. Immunopharmacol.* 78, 106066. doi: 10.1016/j.intimp.2019.106066

Xiong, Y., Yang, J., Tong, H., Zhu, C., and Pang, Y. (2022). HMGB1 augments cognitive impairment in sepsis-associated encephalopathy by binding to MD-2 and promoting NLRP3-induced neuroinflammation. *Psychogeriatrics*. 22(2), 167-179. doi: 10.1111/psyg.12794

Xu, X. E., Liu, L., Wang, Y. C., Wang, C. T., Zheng, Q., and Liu, Q. X., et al. (2019). Caspase-1 inhibitor exerts brain-protective effects against sepsis-associated encephalopathy and cognitive impairments in a mouse model of sepsis. *Brain Behav. Immun.* 80, 859-870. doi: 10.1016/j.bbi.2019.05.038

Xu, Z., Mu, S., Liao, X., Fan, R., Gao, W., and Sun, Y., et al. (2020). Estrogen protects against liver damage in sepsis through inhibiting oxidative stress mediated activation of pyroptosis signaling pathway. *PLoS One*. 15(10), e239659. doi: 10.1371/journal.pone.0239659

Yang, C., Xia, W., Liu, X., Lin, J., and Wu, A. (2019). Role of TXNIP/NLRP3 in sepsis-induced myocardial dysfunction. *Int. J. Mol. Med.* 44(2), 417-426. doi: 10.3892/ijmm.2019.4232

Yang, L., Zhang, H., and Chen, P. (2018). Sulfur dioxide attenuates sepsis-induced cardiac dysfunction via inhibition of NLRP3 inflammasome activation in rats. *Nitric Oxide*. 81, 11-20. doi: 10.1016/j.niox.2018.09.005

Yang, M., Fang, J. T., Zhang, N. S., Qin, L. J., Zhuang, Y. Y., and Wang, W. W., et al. (2021). Caspase-1-Inhibitor AC-YVAD-CMK inhibits pyroptosis and ameliorates acute kidney injury in a model of sepsis. *Biomed Res. Int.* 2021, 6636621. doi: 10.1155/2021/6636621

Yang, W., Tao, K., Zhang, P., Chen, X., Sun, X., and Li, R. (2022). Maresin 1 protects against lipopolysaccharide/d-galactosamine-induced acute liver injury by inhibiting macrophage pyroptosis and inflammatory response. *Biochem. Pharmacol.* 195, 114863. doi: 10.1016/j.bcp.2021.114863

Yu, J., Chen, J., Yang, H., Chen, S., and Wang, Z. (2019). Overexpression of miR200a3p promoted inflammation in sepsisinduced brain injury through ROSinduced NLRP3. *Int. J. Mol. Med.* 44(5), 1811-1823. doi: 10.3892/ijmm.2019.4326

Zhang, B., Liu, Y., Sui, Y. B., Cai, H. Q., Liu, W. X., and Zhu, M., et al. (2015). Cortistatin inhibits NLRP3 inflammasome activation of cardiac fibroblasts during sepsis. *J. Card. Fail.* 21(5), 426-433. doi: 10.1016/j.cardfail.2015.01.002

Zhang, H., Du Y, Guo, Y., Wang, Z., Li, H., and Lv, Z., et al. (2021). TLR4-NLRP3-GSDMD-Mediated pyroptosis plays an important role in aggravated liver injury of CD38(-/-) sepsis mice. *J Immunol Res*. 2021, 6687555. doi: 10.1155/2021/6687555

Zhang, J. L., Chen, Y. T., Chen, G. D., Wang, T., Zhang, J. X., and Zeng, Q. Y. (2017). Glucose-Insulin-Potassium alleviates intestinal mucosal barrier injuries involving decreased expression of uncoupling protein 2 and NLR Family-Pyrin Domain-Containing 3 inflammasome in polymicrobial sepsis. *Biomed Res. Int.* 2017, 4702067. doi: 10.1155/2017/4702067

Zhang, J., Wang, C., Wang, H., Li, X., Xu, J., and Yu, K. (2021). Loganin alleviates sepsis-induced acute lung injury by regulating macrophage polarization and inhibiting NLRP3 inflammasome activation. *Int. Immunopharmacol.* 95, 107529. doi: 10.1016/j.intimp.2021.107529

Zhang, T., Li, J., Liu, C. P., Guo, M., Gao, C. L., and Zhou, L. P., et al. (2021). Butyrate ameliorates alcoholic fatty liver disease via reducing endotoxemia and inhibiting liver gasdermin D-mediated pyroptosis. *Ann Transl Med*. 9(10), 873. doi: 10.21037/atm-21-2158

Zhang, W., Xu, X., Kao, R., Mele, T., Kvietys, P., and Martin, C. M., et al. (2014). Cardiac fibroblasts contribute to myocardial dysfunction in mice with sepsis: The role of NLRP3 inflammasome activation. *PLoS One*. 9(9), e107639. doi: 10.1371/journal.pone.0107639

Zhang, X. P., Zhang, W. T., Qiu, Y., Ju, M. J., Yang, C., and Tu, G. W., et al. (2020). Cyclic helix B peptide alleviates sepsis-induced acute lung injury by downregulating NLRP3 inflammasome activation in alveolar macrophages. *Int. Immunopharmacol.* 88, 106849. doi: 10.1016/j.intimp.2020.106849

Zhang, X. Y., Chen, X., Zhang, H. F., Guan, S., Wen, S. H., and Huang, W. Q., et al. (2018). Propofol does not reduce pyroptosis of enterocytes and intestinal epithelial injury after lipopolysaccharide challenge. *Dig Dis Sci*. 63(1), 81-91. doi: 10.1007/s10620-017-4801-x

Zhang, Z. T., Zhang, D. Y., Xie, K., Wang, C. J., and Xu, F. (2021). Luteolin activates Tregs to promote IL-10 expression and alleviating caspase-11-dependent pyroptosis in sepsis-induced lung injury. *Int. Immunopharmacol.* 99, 107914. doi: 10.1016/j.intimp.2021.107914

Zhang, Z., Han, N., and Shen, Y. (2020). S100A12 promotes inflammation and cell apoptosis in sepsis-induced ARDS via activation of NLRP3 in fl ammasome signaling. *Mol. Immunol.* 122, 38-48. doi: 10.1016/j.molimm.2020.03.022

Zhao, J., Liu, Z., and Chang, Z. (2021). Lipopolysaccharide induces vascular endothelial cell pyroptosis via the SP1/RCN2/ROS signaling pathway. *Eur. J. Cell Biol.* 100(4), 151164. doi: 10.1016/j.ejcb.2021.151164

Zhong, M., Wu, W., Wang, Y., Mao, H., Song, J., and Chen, S., et al. (2020). Inhibition of Sphingosine Kinase 1 Attenuates Sepsis-induced Microvascular Leakage via Inhibiting Macrophage NLRP3 Inflammasome Activation in Mice. *Anesthesiology*. 132(6), 1503-1515. doi: 10.1097/ALN.0000000000003192

Zhong, X., Xie, L., Yang, X., Liang, F., Yang, Y., and Tong, J., et al. (2020). Ethyl pyruvate protects against sepsis-associated encephalopathy through inhibiting the NLRP3 inflammasome. *Mol. Med.* 26(1), 55. doi: 10.1186/s10020-020-00181-3

Zhou, J., Zhang, F., Lin, H., Quan, M., Yang, Y., and Lv, Y., et al. (2020). The protein kinase r inhibitor c16 alleviates Sepsis-Induced acute kidney injury through modulation of the NF-kappaB and NLR family pyrin Domain-Containing 3 (NLPR3) pyroptosis signal pathways. *Med Sci Monit*. 26, e926254. doi: 10.12659/MSM.926254

Zhuo, Y., Yang, L., Li, D., Zhang, L., Zhang, Q., and Zhang, S., et al. (2022). Syringaresinol resisted Sepsis-Induced acute lung injury by suppressing pyroptosis via the oestrogen receptor-beta signalling pathway. *Inflammation*. 45(2), 824-837. doi: 10.1007/s10753-021-01587-9
