# Supplementary figures and images for: Molecular mechanisms and functions of pyroptosis in sepsis and sepsis-associated organ dysfunction
Source: Front Cell Infect Microbiol. 2022 Jul 29;12:962139. doi: 10.3389/fcimb.2022.962139 (PMC9372372; doi:10.3389/fcimb.2022.962139)

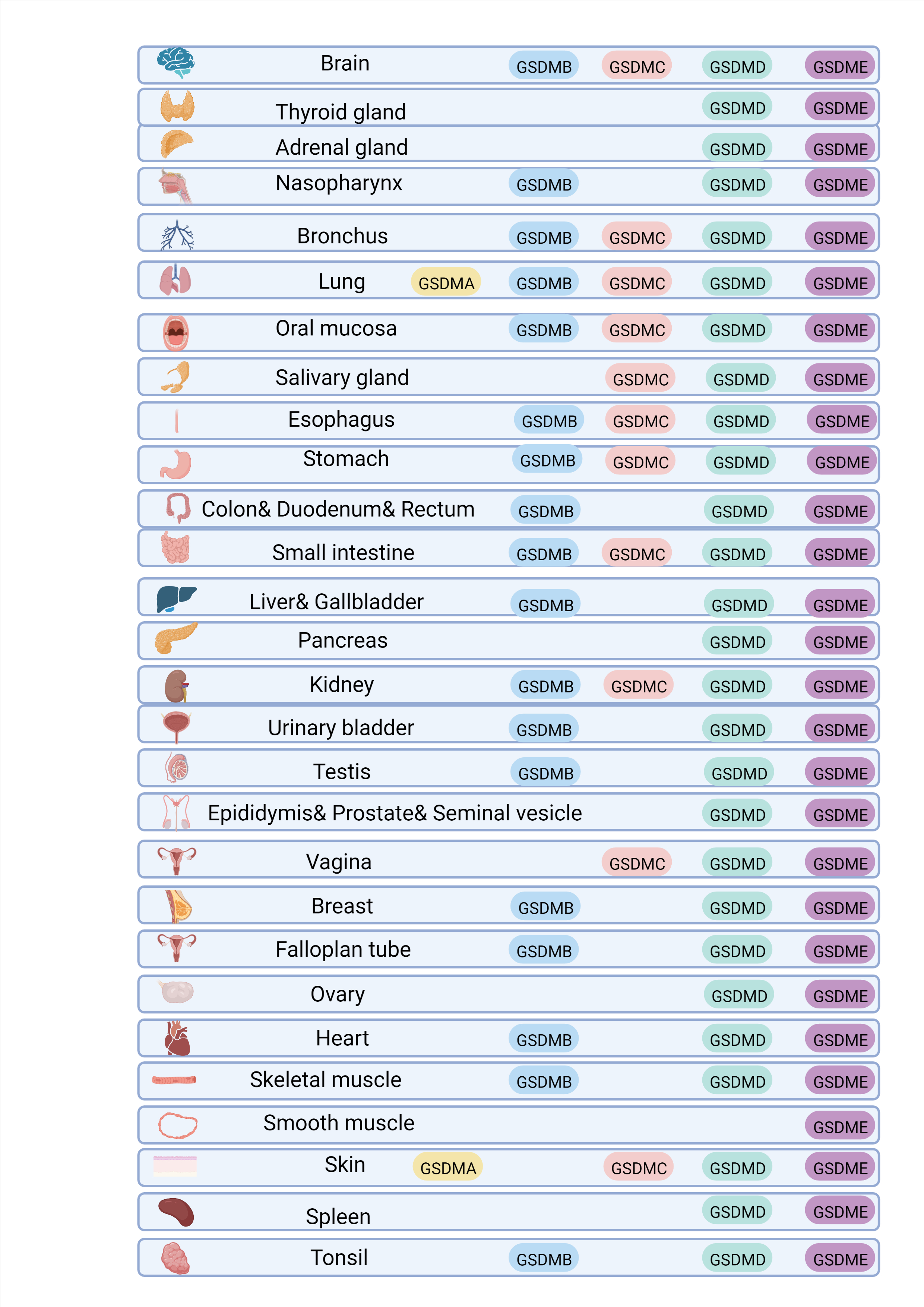

Supplement: Supplementary Figure 1 — Expression of the gasdermins family in different organs. [file Image_1.jpeg]
